# Inter- and Intra-Individual Variability of Personal Health Risk of Combined Particle and Gaseous Pollutants across Selected Urban Microenvironments

**DOI:** 10.3390/ijerph19010565

**Published:** 2022-01-05

**Authors:** Shakhaoat Hossain, Wenwei Che, Alexis Kai-Hon Lau

**Affiliations:** 1 Division of Environment and Sustainability, The Hong Kong University of Science and Technology, Clear Water Bay, Kowloon, Hong Kong; mshossain@connect.ust.hk (S.H.); alau@ust.hk (A.K.-H.L.); 2 Department of Public Health and Informatics, Jahangirnagar University, Dhaka 1342, Bangladesh; 3 Department of Civil and Environmental Engineering, The Hong Kong University of Science and Technology, Clear Water Bay, Kowloon, Hong Kong

**Keywords:** air pollution, personal exposure concentration, health risk, variability, microenvironments

## Abstract

Exposure surrogates, such as air quality measured at a fixed-site monitor (FSM) or residence, are typically used for health estimates. However, people spend various amounts of time in different microenvironments, including the home, office, outdoors and in transit, where they are exposed to different magnitudes of particle and gaseous air pollutants. Health risks caused by air pollution exposure differ among individuals due to differences in activity, microenvironmental concentration, as well as the toxicity of pollutants. We evaluated individual and combined added health risks (AR) of exposure to PM_2.5_, NO_2,_ and O_3_ for 21 participants in their daily life based on real-world personal exposure measurements. Exposure errors from using surrogates were quantified. Inter- and intra-individual variability in health risks and key contributors in variations were investigated using linear mixed-effects models and correlation analysis, respectively. Substantial errors were found between personal exposure concentrations and ambient concentrations when using air quality measurements at either FSM or the residence location. The mean exposure errors based on the measurements taken at either the FSM or residence as exposure surrogates was higher for NO_2_ than PM_2.5_, because of the larger spatial variability in NO_2_ concentrations in urban areas. The daily time-integrated AR for the combined PM_2.5_, NO_2_, and O_3_ (TIAR_combine_) ranged by a factor of 2.5 among participants and by a factor up to 2.5 for a given person across measured days. Inter- and intra-individual variability in TIAR_combine_ is almost equally important. Several factors were identified to be significantly correlated with daily TIAR_combine_, with the top five factors, including PM_2.5_, NO_2_ and O_3_ concentrations at ‘home indoor’, O_3_ concentrations at ‘office indoor’ and ambient PM_2.5_ concentrations. The results on the contributors of variability in the daily TIAR_combine_ could help in targeting interventions to reduce daily health damage related to air pollutants.

## 1. Introduction

Adverse health effects of air pollution are a major public health issue globally. The World Health Organization (WHO) estimated that over 80% of the population living in urban areas breathes where the air quality does not meet the WHO guidelines [1]. Exposure to air pollution is related to numerous types of health damage. Exposure to daily PM_2.5_ concentrations has a causal relationship with cardiovascular illnesses and premature mortality [2]. There is a causal relationship between short-term exposure to 1 h NO_2_ concentrations and respiratory disease outcomes [3]. Exposure to an 8 h daily maximum ambient O_3_ concentration is causally associated with respiratory illness [4].

A number of epidemiological studies have estimated the health risk of air pollution based on fixed-site monitor (FSM) as a surrogate for exposure because of readily available real-time data with high measurement accuracy [5,6,7,8,9]. People typically spend over 85 percent of their time in indoor environments where they are usually exposed to different levels of concentrations than ambient concentrations [10]. For example, Che et al. [11] demonstrated that, at school, the mean exposure error from using outdoor PM_2.5_ concentrations at FSM as a surrogate was up to 30%. Some studies estimated exposure based on static residence-based approach (e.g., land use regression approach) to model people’s exposure to air pollution using nearby monitoring stations at residential addresses [12,13,14,15]. However, exposure concentration varies among microenvironments (e.g., ‘home indoor’, ‘office indoor’, ‘others indoor’, ‘outdoor’ and ‘transit’) [16,17]. For example, Koehler et al. [17] demonstrated that PM_2.5_ exposure concentrations substantially varied across different microenvironments with a geometric mean of 8.0 µg/m^3^ at home, 2.4 µg/m^3^ at work, 6.8 µg/m^3^ in transit, 8.4 µg/m^3^ at an eatery and 4.9 µg/m^3^ in other microenvironments. The residence-based approach is unable to capture the variation in air pollution exposure across different microenvironments because it ignores human mobility and does not properly apprehend the space-time dynamics of air pollution [18]. Thus, the residence-based approach as a surrogate may lead to a bias in the exposure-response relationship. Little is known about the exposure error between a surrogate and real-world exposure concentrations.

The health risk of exposure to air pollution depends on the exposure concentration over time in a microenvironment that leads us to quantify time-integrated or personal exposure across the microenvironments [19]. Personal exposure to air pollution is defined as the product of the average personal exposure concentration in a microenvironment and the total time spent in that microenvironment [16]. To date, some studies quantified the variability of personal exposure to air pollution across different microenvironments for a single pollutant [16,20,21,22]. For example, Mazaheri et al. [16] investigated the variability of personal PM_2.5_ exposure across different microenvironments and activities among 14 adult participants in Queensland, Australia. However, people are typically exposed to a mixture of particle and gaseous pollutants simultaneously in the real world. Each air pollutant is different in terms of health risk and toxicity, which leads us to quantify personal exposure to combined particle and gaseous pollutants. To our best knowledge, no study has been conducted to quantify overall exposure variability for combined personal PM_2.5_, NO_2_ and O_3_ exposure concentrations across the microenvironments to date.

Because an individual’s mobility varies in location and time, exposure concentrations may differ widely between individuals and over time for a given individual [23,24,25]. Previous studies have demonstrated the inter- and intra-individual variability in personal exposure concentrations for single pollutants [25,26,27,28]. In Guangzhou, China, variance component analysis demonstrated that the intra-individual variability was 90% in the daily lognormal personal PM_2.5_ exposure concentrations measured in 16 adult subjects [26]. In contrast, Chen et al. [27] reported that inter- and intra-individual variability in personal PM_2.5_ exposure concentration were almost equal. For NO_2_, Lee et al. [29] demonstrated that the intra-individual variability was higher than inter-individual variability in daily exposure concentrations. Grivas et al. [30] reported that the inter- and intra-individual variability were almost similar (51–56% intra-individual variability across the cities) in weekly average O_3_ exposure concentrations among school children in two cities, Athens and Thessaloniki, in Greece. However, because people are exposed to multiple pollutants simultaneously, it is important to quantify inter- and intra-individual variability and the key contributors of variability in the combined exposure to PM_2.5_, NO_2_ and O_3_ concentrations. No information is available on the inter- and intra-individual variability in the combined exposure to PM_2.5_, NO_2_ and O_3_, and the key contributors affecting the variability. Identifying and characterizing the key contributors of inter- and intra-individual variability in combined PM_2.5_, NO_2_ and O_3_ exposure may help in targeting interventions to reduce the health effects of exposure to a mixture of air pollutants.

The concentrations of different pollutants cannot be added directly to quantify combined exposure due to their differences in sampling units and their toxicity in terms of health risks. To overcome this limitation, an added health risk (AR) model had been applied in this study which was developed by Wong et al. [31] as the basis of the multipollutant air quality health index (AQHI), considering local hospital admissions data for the 3 h moving average of individual air pollutants. The AQHI approach is used by the Hong Kong Environmental Protection Department (HKEPD) to provide advice to the public regarding short-term health risks of air pollution [32] and was also used in our previous study to evaluate the impacts of ambient concentration changes due to emission control measures in Hong Kong [33]. In this study, we would use the same method to combine the health risks from multiple pollutants and assess the inter- and intra- individual variability in terms of the time-integrated health risk.

The objectives are to: (1) quantify the exposure error for PM_2.5_, NO_2_ and O_3_ between personal exposure concentrations across the selected microenvironments and ambient concentration from the FSM and residence location; (2) quantify the time-integrated AR (TIAR) for the combined PM_2.5_, NO_2_ and O_3_ exposure concentrations for each participant on each measured day and its contributions from selected microenvironments; and (3) identify key contributors of inter- and intra-individual variability in TIAR.

## 2. Materials and Methods

This section describes: the study design for personal exposure concentration measurements; the instrumentation for air quality measurements; the time-location record of the participants; the exposure error estimation between personal exposure concentrations and ambient concentrations; the calculation of health risk; time-integrated health risk; and inter- and intra-individual variability in TIAR for PM_2.5_, NO_2_, and O_3_ and combined PM_2.5_, NO_2_, and O_3_ exposure concentrations.

The supplementary material contains additional details on: (a) sensors specifications that are used to measure personal exposure concentrations; (b) carbon dioxide as an indicator of microenvironments to check the consistency of the recorded time-location patterns of the individual; (c) sensitivity analysis of AR estimation based on 1 min exposure concentrations of the pollutants instead of 3 h moving average concentrations; and (d) quantification of inter- and intra-individual variability in the daily time spent in each microenvironment.

### 2.1. Study Design

To quantify the health risk of combined personal exposure to particle and gaseous pollutants across the selected microenvironments, a longitudinal study was conducted among twenty-one participants. All participants were recruited from faculty, staff, and postgraduate students from the Hong Kong University of Science and Technology, Hong Kong. The participants lived in different districts and areas across Hong Kong, including urban and suburban areas. The air quality of Hong Kong is influenced by local emission sources, including on-road motor vehicles, power plants and marine vessels [33], and regional air pollution from mainland China [34]. Each participant performed the continuous air quality measurement on multiple consecutive days based on their availability, with an average of five days. Data were collected for 106 person-days. During the personal monitoring campaigns, the outdoor air quality of residences was also measured for seven participants. Demographic information (e.g., age, occupation, number of occupants at home, living floor, smoking) and residential characteristics (e.g., type of ventilation status at home, AC type, type of cooking stoves, cooking duration per day and frequency of floor cleaning) were collected using a web-based structured questionnaire.

### 2.2. Instrumentation

**Figure 1:**
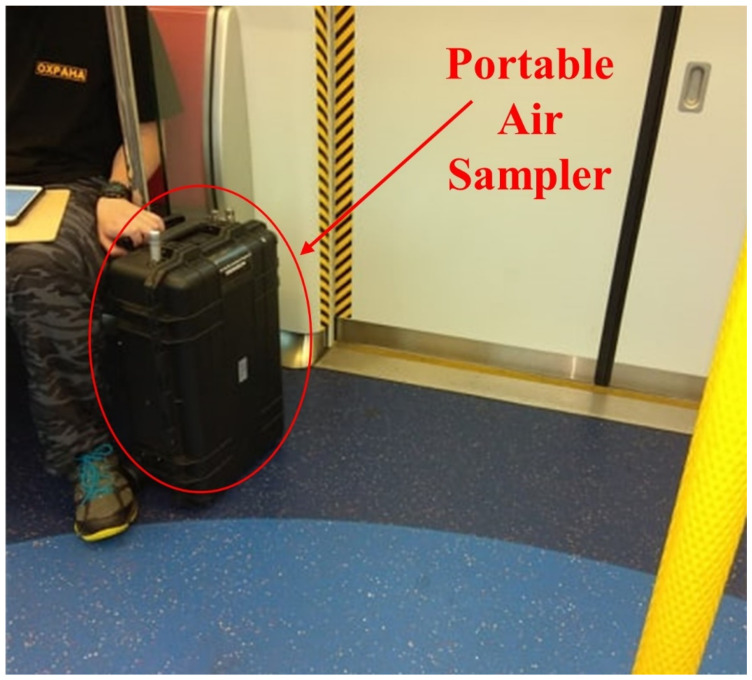
Real-time (24 h) personal air quality measurement using portable air sampler.

The PM_2.5_, NO_2_, and O_3_ exposure concentration were measured across the microenvironments that were visited by the participants. As an indicator of indoor and outdoor differences, CO_2_ concentrations were also measured. PM_2.5_ concentrations were measured using a photometer Aerocet 831 Handheld Particle Counter (Metone, Grants Pass, OR, USA) for personal exposure concentration across the microenvironments and a photometer model 212 Ambient Particulate Profiler (Metone, Grants Pass, OR, USA) for outdoor air quality at residence locations. The concentrations of O_3_ and NO_2_ were measured using electrochemical sensors that were the model B series from Alphasense, Braintree, UK. The CO_2_ concentration was measured using a nondispersive infrared Premier CO_2_ sensor from Dynament, Mansfield, UK. The sensors of all pollutants were integrated into a portable system called Portable Air Station (PAS) and an outdoor portable system called a Mini Air Station (MAS) [35,36]. The PAS was used to measure personal exposure concentrations across the microenvironments (Figure 1). The PAS was able to store local data and transmit data to a server in real-time.

The sensors measured air pollution concentrations at 1 min resolution. The PAS has 12 V Li-ion Battery inside for a standby capacity of more than 10 h. The PAS has rolling wheels that make the 15 kg PAS convenient to move from one place to another with participants. Participants were instructed to always carry the instrument and keep the instrument within 1 m of them in indoor environments (e.g., home and office). Participants were given some flexibility to keep the PAS in a nearby safe place while taking a bath.

Details of the quality assurance (QA) and quality control (QC) for the sensor measurements are provided elsewhere [36,37,38]. The accuracy of the sensors used in PAS and MAS were evaluated based on measurements with standard gas at known concentrations in the laboratory and collocation with reference methods at the government operated Air Quality Monitoring Station (AQMS) at Mong Kok. The differences in concentration measurements between the reference methods and sensors were, on average, less than 1% for PM_2.5_ and NO_2_, and about 4% for O_3_ over 350 sampling hours [38]. During air quality measurements and post data analysis, QA and QC were maintained to obtain a high quality of data. Detailed procedures regarding the QA and QC for air quality data are published elsewhere [39].

### 2.3. Time-Location Records

Participants recorded the start and end times of each location that they visited and categorized each location into one of the five predefined microenvironment categories using the aTimeLogger app on their smartphone (Available online: http://atimelogger.com, accessed on 20 December 2021). The five categories of microenvironments were: ‘home indoor’, ‘office indoor’, ‘others indoor’, ‘outdoor’, and ‘transit’. Two participants recorded their time-location information in open text on their smartphones. Participants were asked to record their time-location immediately after changing each microenvironment to avoid misclassification of time-location information.

CO_2_ is produced by human exhalation as a function of metabolic processes [40]. The outdoor CO_2_ concentration level is lower than the CO_2_ concentration in indoor environments because of human occupancy [41]. Based on our analysis, CO_2_ concentration substantially varied between indoor and outdoor microenvironments and among different indoor microenvironments depending on the occupancy number and ventilation condition, provided in supplementary material. Therefore, we used real-time CO_2_ concentration time-series (1 min resolution) in this study to check the consistency of the recorded time-location patterns of the individual.

### 2.4. Quantification of Exposure Error between Personal Exposure Concentrations and Ambient Concentrations

To compare personal exposure concentrations with the ambient concentration at the residence location, the outdoor air quality was measured at seven participants’ residences. The MAS was used to measure residences’ outdoor air quality. The MAS was placed on the balcony or another outdoor space of the residence.

Hourly-average ambient concentrations of PM_2.5_, NO_2_ and O_3_ were retrieved from FSM stations of the Hong Kong Environmental Protection Department (HKEPD). FSM stations were selected based on the nearest distance to the participant’s resident address.

The exposure error between personal exposure concentrations and ambient concentrations was estimated as below:

(1) $${\mathrm{EE}}_{p,j,i}=\left|\frac{{\mathrm{PC}}_{p,j,i}-{\mathrm{AC}}_{p,j,i,l\;}}{{\mathrm{PC}}_{p,j,i}}\right|\times 100$$

where EE_p,j,i_ is the exposure error (%) in daily average concentration between personal exposure concentration and ambient concentration of pollutant p in each person-day j for participant i. PC_p,j,i_ is the daily average personal exposure concentrations of pollutant p in each person-day j for participant i. AC_p,j,i,l_ is the daily average ambient concentrations of pollutant p in each person-day j for participant i at location l (i.e., nearest FSM or residence location).

### 2.5. Calculation of Health Risk

For a mixture of PM_2.5_, NO_2_ and O_3_, AR of hospital admission for respiratory diseases (International Classification of Disease codes 460-519) and cardiovascular diseases (International Classification of Disease codes 390-459) for all ages were quantified [31]. Although AR in AQHI was quantified hourly based on a 3 h moving average of ambient concentrations [31,32], we estimated AR based on continuous 1 min personal exposure concentrations. This is because we quantified AR in each microenvironment across the person-days, and participants spent less than 3 h in some of the microenvironments. Thus, 3 h moving average concentrations may cause misrepresentation of those microenvironments by averaging before and after the microenvironmental concentrations. Based on our analysis, the median differences of the daily average AR estimation between 1 min exposure concentrations and 3 h moving average concentrations were less than 2% for each pollutant, regardless of the microenvironments. For each pollutant (AR_p_) in the mixture, AR is quantified:

AR_p_ = [exp(β_p_.C_pt_) − 1] 100% (2)

where C_pt_ is the 1 min personal exposure concentration (µg/m^3^) of pollutant p at time t, and β_p_ is a regression coefficient in units of (µg/m^3^)^−1^ of pollutant p. Wong et al. [31] reported the values of β_p_ based on time series analysis, including β_PM2.5_ = 2.1 × 10^−4^ per µg/m^3^, β_NO2_ = 4.5 × 10^−4^ per µg/m^3^ and β_O3_ = 5.1 × 10^−4^ per µg/m^3^.

The AR for a mixture of PM_2.5_, NO_2_, and O_3_ is quantified:

AR_combine_ = AR_PM2.5_ + AR_NO2_ + AR_O3_ (3)

where AR_combine_ is the sum of the AR for PM_2.5_, NO_2_ and O_3_ concentrations

### 2.6. Time-Integrated Health Risk

Because the health risk of air pollution depends on the exposure concentration over time in a microenvironment, the contribution of each microenvironment to the daily health risk was estimated based on TIAR. The TIAR for a mixture of PM_2.5_, NO_2_ and O_3_ across the microenvironments for a participant was estimated as

(4) $${\mathrm{TIAR}}_{\mathrm{combine},k,i,j}=\sum_{i}^{n}{\mathrm{AR}}_{\mathrm{combine},\;k,i,j}t_{k,i,j}\;$$

where, ${\mathrm{TIAR}}_{\mathrm{combine},k,i,j}$ is time-integrated AR_combine_ (%h) in microenvironment k in person-day j exposed by participant i for the AR of combined PM_2.5_, NO_2_ and O_3_; ${\mathrm{AR}}_{\mathrm{combine},\;k,i,j}$ is the average AR_combine_ (%) in microenvironment k for participant i in person-day j; $t_{k,i,j}$ is the duration (hour) spent in microenvironment k by participant i in person-day j; and n is the total number of microenvironments. This approach of estimating the TIAR in a given microenvironment is similar to the method for estimating time-integrated exposure in a microenvironment used by Klepeis [42].

We also quantified TIAR across the microenvironments for the individual pollutant, i.e., PM_2.5_, NO_2_ and O_3_ exposure concentrations:

(5) $${\mathrm{TIAR}}_{p,k,i,j}=\sum_{i}^{n}{\mathrm{AR}}_{p,\;k,i,j}t_{k,i,j}$$

where, ${\mathrm{TIAR}}_{p,k,i,j}$ is time-integrated AR_p_ (%h) in microenvironment k in person-day j exposed by participant i for the pollutant p; ${\mathrm{AR}}_{p,\;k,i,j}$ is the average AR_p_ (%) in microenvironment k for participant i in person-day j for pollutant p.

To quantify which microenvironments contribute disproportionately, the health risk relative intensity was estimated. Health risk intensity was calculated by dividing the fraction of the health risk for a participant exposed in a microenvironment by the time fraction of the day that the participant spent in that microenvironment. The approach of calculating health risk intensity is similar to the approach for calculating the dose relative intensity estimation used by Mazaheri et al. [43].

(6) $$I_{k,j,i}=\frac{\frac{{\mathrm{TIAR}}_{\mathrm{combine},k,j,i}}{\sum_{j}{\mathrm{TIAR}}_{\mathrm{combine},j,i}}\times 100}{\frac{t_{k,j,i}}{\sum_{j}t_{j,i}}\times 100}$$

where, $I_{k,j,i}$ is the health risk intensity in microenvironment k in person-day j for participant i; ${\mathrm{TIAR}}_{\mathrm{combine},j,i}$ is the total time-integrated AR_combine_ (%h) on a given day of j for participant i; $t_{k,j,i}$ is total time spent (hour) in microenvironment k in person-day j for participant i; and $t_{j,i}$ is total spent on a given day of j for participant i. A relative intensity value greater than one for a microenvironment suggests that the fraction of the TIAR_combine_ was higher than the time spent in that microenvironment. A value smaller than one indicates that the fraction of the TIAR_combine_ was less than the fraction of the time spent in that microenvironment.

### 2.7. Quantifying Inter- and Intra-Individual Variability in Time-Integrated Health Risk

To quantify proportions of inter- and intra-individual variability in the daily total TIAR, variance component analysis was used. Variance component analysis in the daily total TIAR was performed separately for PM_2.5_, NO_2_ and O_3_, and combined PM_2.5_, NO_2_ and O_3_. To quantify the variance component, a linear mixed-effects model with only a random intercept was developed [17,25]:

Z_ij_ = µ + b_i_ + ε_ij_ (7)

where Z_ij_ is the log-transformed daily total TIAR for PM_2.5_, NO_2_ and O_3_, and combined PM_2.5_, NO_2_ and O_3_ in person-day j for participant i; μ is the fixed mean (logged) TIAR for all subjects; and ε_ij_ is the error. In linear mixed-effects models, a person-specific random effect b_i_ assumed to be normally distributed with zero mean and variance $\sigma_{\mathrm{inter}}^{2}$ (the inter-individual variability). The error ε_ijp_ is assumed to have normal distribution with zero mean and variance $\sigma_{\mathrm{intra}}^{2}$ (the intra- variability). The variance components for inter- and intra-individual were estimated using the method of restricted maximum likelihood (REML) [44].

Intra-class correlation coefficients (ICC), i.e., the proportion of total variability attributable to inter-individual variability, was estimated as [45]:

(8) $$\mathrm{ICC}=\sigma_{\mathrm{inter}}^{2}/(\sigma_{\mathrm{intra}}^{2}+\sigma_{\mathrm{inter}}^{2})$$

To identify key contributors affecting the inter- and intra-individual variability in daily TIAR_combine_, we performed correlation analysis between TIAR_combine_ and selected independent variables. The variables were the daily time spent at ‘home indoor’, ‘office indoor’, ‘others indoor’, ‘outdoor’, and ‘transit’; the daily average PM_2.5_ concentrations at ‘home indoor’, ‘office indoor’, ‘others indoor’, ‘outdoor’, and ‘transit’; the daily average NO_2_ concentrations at ‘home indoor’, ‘office indoor’, ‘others indoor’, ‘outdoor’, and ‘transit’; the daily average O_3_ concentrations at ‘home indoor’, ‘office indoor’, ‘others indoor’, ‘outdoor’, and ‘transit’; and the daily average ambient PM_2.5_, NO_2_ and O_3_ concentrations. The variables were averaged daily and for each participant in order to analyze correlation with the daily TIAR_combine_ and participant-wise average TIAR_combine_, respectively.

### 2.8. Statistical Analysis

The normality of the distribution of data was checked using the Shapiro–Wilks test. For non-normal distribution, the Mann–Whitney test was used to determine the statistical significance of the difference between two independent groups. The Kruskal–Wallis test was used to compare more than two independent groups.

## 3. Results

The results include study participants and the time-location pattern; exposure error between personal exposure concentrations and ambient concentrations; time-integrated health risk across the selected microenvironments; and inter-individual and intra-individual variability in the time-integrated health risk.

The supplementary material contains additional results for (a) general characteristics of the study participants; (b) inter- and intra-individual variability in the daily time spent by participants in each microenvironment; (c) summary statistics of personal exposure concentrations of PM_2.5_, NO_2_ and O_3_ in each microenvironment across the participants; and (d) the relationship of ambient PM_2.5_, NO_2_ and O_3_ concentrations between FSM and the residence location.

### 3.1. Study Participants and Time-Location Pattern

General characteristics of the participants are given in the supplementary material. Twenty-one participants aged 21–60 years old participated in the longitudinal measurement of the health risk of personal exposure study. All of the participants are non-smokers and lived in non-smoking homes.

The mean daily percent of time spent by participants across the microenvironments are provided in Table 1. Participants spent, on average, only 2.5% of their daily time in an ‘outdoor’ environment. Among the five categories of microenvironments, participants spent most of their daily average time in the ‘home indoor’ (range: 9.3–24.0 h), followed by the ‘office indoor’ (range: 0–13.9 h), the ‘others indoor’ (range: 0–7.5 h), in transit (range: 0–5.8 h) and in the ‘outdoor’ (range: 0–4.6 h) microenvironment. Based on variance component analysis (given in supplementary material), intra-individual variability in daily time spent in all selected microenvironments ranged from 59% to 91%. Inter-individual variability was from 9% to 41% across the microenvironments. This indicates that the day to day variability in the time spent for a given individual in a microenvironment is higher than the differences in the average time spent in that microenvironment between individuals during the whole measurement period.

### 3.2. Exposure Error between Personal Exposure Concentrations and Ambient Concentration

**Figure 2:**
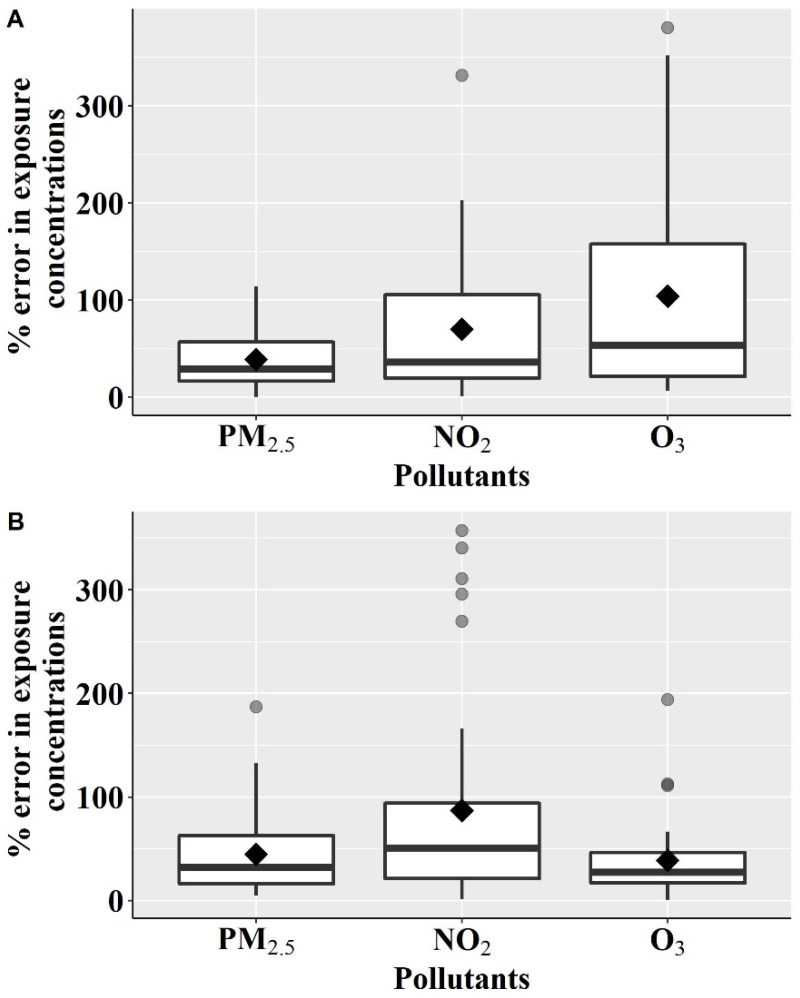
Percentage error in daily average exposure concentrations of PM_2.5_, NO_2_ and O_3_ between personal exposure concentrations across the different microenvironments and ambient concentrations: (**A**) ambient concentrations from fixed-site monitors nearby the participant’s residence (N = 106); (**B**) ambient concentrations at residence location (N = 35).

The error percentage between the daily average personal exposure concentrations and ambient concentration for PM_2.5_, NO_2_ and O_3_ are provided Figure 2, where Figure 2A indicates the ambient concentrations from FSM and Figure 2B indicates the ambient concentrations from the residence location.

When ambient concentrations were based on FSM, the mean error percentage was 39% and 70% for PM_2.5_ and NO_2_ concentrations, respectively. In comparison, the mean errors were 45% for PM_2.5_ and 87% for NO_2_ when ambient concentration was based on the residence location. This exposure error is primarily because of the spatial variability of the concentrations due to the daily movement of the participants across the microenvironments. The mean exposure error of NO_2_ was higher than PM_2.5_ when based on either the measurements at FSM and residence as exposure surrogates because of larger the spatial variability in NO_2_ concentrations in urban areas. The coefficient of determination (R^2^) values between daily average concentrations at FSM and the residence location was lower for NO_2_ (0.25) than PM_2.5_ (0.87) (this is provided in the supplementary material), indicating the larger spatial variability of NO_2_ concentrations.

For O_3_, the mean exposure error was 104% based on the air quality measurements at FSM and 39% based on the measurements at the residence location. This is because people are exposed to lower levels of personal exposure concentrations in different microenvironments than outdoors. For example, on a given person-day, the average personal O_3_ exposure concentrations were 27 µg/m^3^ in ‘home indoor’, 37 µg/m^3^ in ‘office indoor’, 25 µg/m^3^ in ‘others indoor’, and 17 µg/m^3^ in ‘transit’, while the daily average ambient O_3_ concentration on that day was 61 µg/m^3^ at FSM. The mean O_3_ exposure error using FSM was higher than using the residence location. This is because FSM used in our study is general FSM located at the top of the roof and not adjacent to busy roads. Thus, we measured higher O_3_ concentrations at FSM. In contrast, the participants live in urban areas close to busy roads, where an abundance of nitric oxide (NO) from on-road vehicles titrate O_3_ concentrations [33]. Thus, lower O_3_ concentrations were measured at residence locations.

The exposure errors based on FSM or residence locations were consistent for PM_2.5_ and lower than 50%, indicating small spatial variability in PM_2.5_ concentrations, similar to the previous study [46,47]. For NO_2_, exposure errors were higher (≥70%) because of larger spatial variability and were consistent throughout, based on FSM and residence locations. This indicates that NO_2_ exposure concentrations based on a single location either FSM or residence location cannot represent the large spatial variability in personal exposure concentrations across the microenvironments. For O_3_, exposure errors were inconsistent between FSM and residence locations because of O_3_ titration by NO. Thus, the results of exposure errors in NO_2_ and O_3_ concentrations as a surrogate highlights the importance of real world personal exposure concentration measurements with the daily active lifestyle of the individuals.

### 3.3. Contribution of the Selected Microenvironments in Time-Integrated Health Risk

The mean percent of the daily time-integrated health risk for combined PM_2.5_, NO_2_ and O_3_ exposure concentrations in each selected microenvironment are given in the supplementary material. There were significant differences (*p* < 0.001) in the median TIAR_combine_ among the microenvironments. The daily highest TIAR_combine_ (mean ± standard deviation, SD) 64% (±19%) was in the ‘home indoor’ microenvironment, where participants spent an average 66% (±17%) of their total daily time. The daily minimum TIAR_combine_ 5% (±5%) was in the ‘outdoor’ microenvironment, where participants spent an average of 3% (±3%) of their daily time. Although AR_combine_ in ‘home indoor’ microenvironments was lower than the ‘others indoor’, ‘outdoor’ and ‘transit’ microenvironments, TIAR_combine_ in ‘home indoor’ were found to be higher than those microenvironments due to the long dwelling time of the participants in the ‘home indoor’ microenvironment.

**Figure 3:**
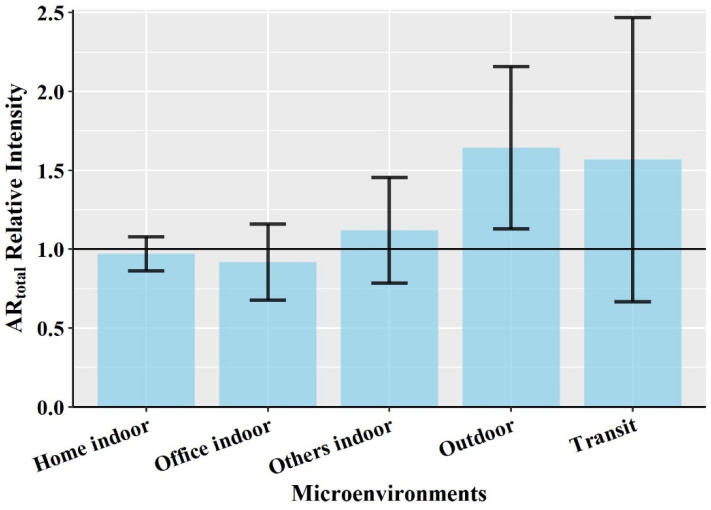
Average time-weighted short-term health risk intensities in each microenvironment. Error bar represents standard deviation (Sample size, home indoor = 106, office indoor = 86, others indoor = 82, outdoor = 85 and transit = 78).

Because TIAR_combine_ is related to the duration spent in each microenvironment, health risk intensities provide a clear picture of which microenvironment contributes disproportionately to daily health risk. Figure 3 presents the average health risk intensities in each microenvironment.

The maximum average health risk intensity 1.64 (±0.52) was found in ‘outdoor’ followed in order by ‘transit’, ‘others indoor’, ‘home indoor’ and ‘office indoor’ microenvironments. Among the microenvironments, intensity values exceeded 1 for the ‘outdoor’, ‘transit’ and ‘others indoor’, which indicated the fraction of TIAR_combine_ in those microenvironments was higher than the faction of time spent in the corresponding microenvironments. This means that, although participants spent only a small fraction of their time in these settings, these microenvironments contribute disproportionately to AR_combine_ because of the elevated concentrations of pollutants. Thus, these microenvironments with large health risk intensity could help to prioritize interventions to reduce the daily health risk. Higher health risk intensity for combined PM_2.5_, NO_2_ and O_3_ exposure in the ‘transit’ microenvironment than in the ‘home indoor’ and ‘others indoor’ was because of the much higher NO_2_ concentrations in the vehicle’s cabin. Although PM_2.5_ and O_3_ concentrations in ‘transit’ were lower than ‘home indoor’ and ‘others indoor’ microenvironments, the higher health risk posed by NO_2_ offset the lower health risk of PM_2.5_ and O_3_ concentration. Among the two main microenvironments, ‘home indoor’ and ‘office indoor’ where participants spend most of their daily time, health risk intensity in the ‘office indoor’ microenvironment was much less than in the ‘home indoor’ microenvironment. This is because the average concentration for PM_2.5_ and NO_2_ in ‘office indoor’ microenvironments were always encountered as lower than the ‘home indoor’ microenvironment. The ‘office indoor’ was operated with a centralized mechanical ventilation and air-conditioned system with high-efficiency filters for particles (Minimum Efficiency Reporting values of 13, MERV 13), whereas the ‘home indoor’ microenvironment was commonly ventilated by opening windows which introduce more infiltration of outdoor pollution than the office. In addition, home is intensely influenced by indoor emission sources, including cooking and cleaning inside of the home. For example, Baxter et al. [48] found that cooking for more than an hour per day was significantly associated with a 5.7 µgm^−3^ increase in indoor PM_2.5_ concentrations compared to less than an hour per day.

### 3.4. Inter-Individual and Intra-Individual Variability in Time-Integrated Health Risk

**Figure 4:**
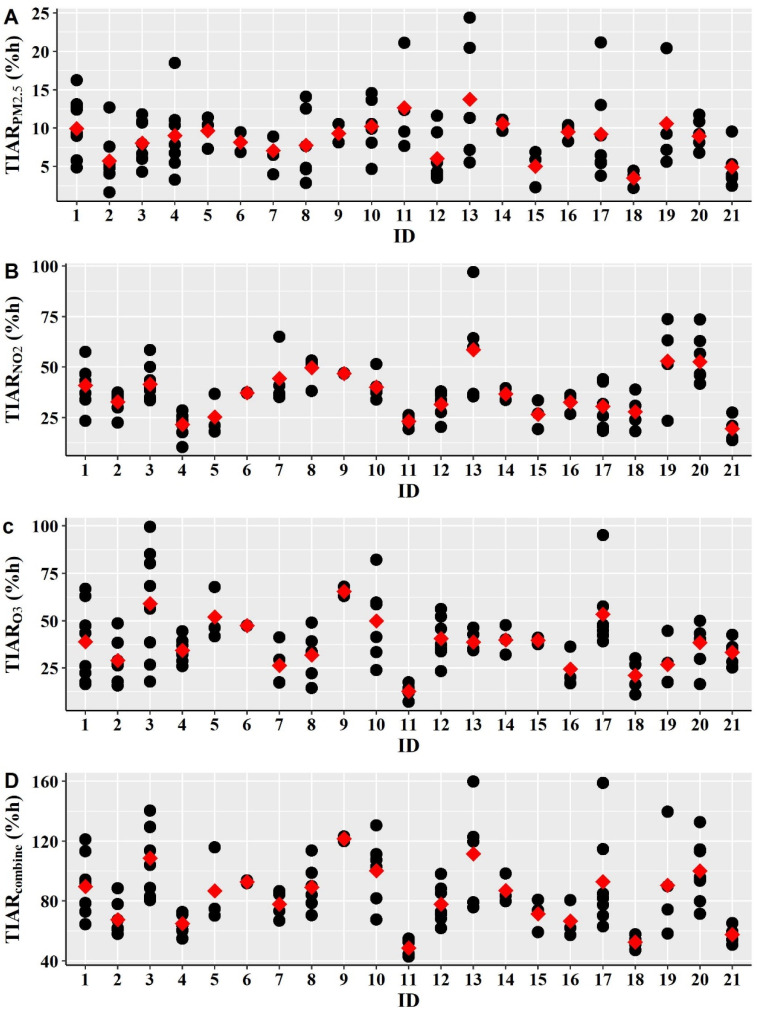
Inter and intra-individual variability of the daily time-integrated health risk (TIAR) for (**A**) PM_2.5_; (**B**) NO_2_; (**C**) O_3_; and (**D**) combined PM_2.5_, NO_2_ and O_3_ exposure concentrations across the selected microenvironments. Black circle represents the total TIAR across the selected microenvironments in each person-day. Red square indicates mean of the TIAR for a participant.

Inter- and intra-individual variability of daily TIAR for PM_2.5_, NO_2_ and O_3_ and combined PM_2.5_, NO_2_ and O_3_ exposure concentrations across the microenvironment is given in Figure 4.

The results demonstrated that there was substantial inter-individual variability in mean TIAR by a factor of 3.9 for TIAR_PM2.5_, 3.0 for TIAR_NO2_, 5.1 for TIAR_O3_ and 2.5 for TIAR_combine_ across the participants. Intra-individual variability indicates the daily fluctuations of TIAR that vary temporally from day to day. Large intra-individual variability in TIAR_PM2.5_, TIAR_NO2_, TIAR_O3_ and TIAR_combine_ was found in many participants. For example, the daily total TIAR_combine_ varied by a factor up to 2.5 across the person-days of a given person. TIAR_PM2.5_, TIAR_NO2_ and TIAR_O3_ varied by a factor of up to 7.8, 3.1 and 5.6 for a given person across the measured days, respectively.

Table 2 shows the variance component for inter- and intra-individual variability in lognormal daily TIAR for each pollutant and for combined PM_2.5_, NO_2_ and O_3_. The inter- and intra-individual variability between particle and gaseous pollutants was substantially different from each other. For TIAR_PM2.5_, intra-individual variability (78%) was substantially higher than inter-individual variability (22%). This indicates that the daily personal activities and lifestyle of the participants are more important than where the participants live because of the small spatial variability in PM_2.5_ concentration. Thus, an individual has the potential to reduce PM_2.5_-induced health risk by modifying their daily activities and lifestyle. In contrast, for gaseous pollutants, inter- and intra-individual variance in both TIAR_NO2_ and TIAR_O3_ were comparable. Because of the larger spatial variability of gaseous pollutants than PM_2.5_, where participants live is important to reduce health risk for NO_2_ and O_3_ exposure. The larger inter-individual in TIAR_NO2_ indicates that the health risk and sources of NO_2_ concentrations are widely varied across the participants. For TIAR_combine_, inter- and intra-individual variability is almost equally important. Thus, for combined PM_2.5_, NO_2_ and O_3_ exposure, both daily personal activities and a participant’s location are important to reduce health damages of air pollution exposure.

Identified key contributors affecting inter- and intra-individual variability in TIAR_combine_ are provided in Table 3. For inter-individual variability, TIAR_combine_ was significantly (*p* < 0.05) correlated with PM_2.5_, NO_2_ and O_3_ exposure concentration at ‘home indoor’ microenvironments, PM_2.5_ and NO_2_ at ‘others indoor’ microenvironments, O_3_ at ‘office indoor’ microenvironments and ambient PM_2.5_ concentrations. Based on an interpretation of the correlation coefficient by Schober et al. [49], exposure to O_3_ concentration at ‘home indoor’ microenvironments was strongly correlated (r = 0.74, *p* < 0.001) with TIAR_combine_. There was a moderate correlation (i.e., 0.45 to 0.60, *p* < 0.05) of TIAR_combine_ with PM_2.5_ and NO_2_ concentration at ‘home indoor’ microenvironments, PM_2.5_ and NO_2_ at ‘others indoor’ microenvironments, O_3_ at ‘office indoor’ microenvironments and ambient PM_2.5_ concentrations.

For intra-individual variability, TIAR_combine_ was strongly correlated (r = 0.76, *p* < 0.001) with O_3_ concentration at ‘home indoor’ microenvironments and moderately correlated (i.e., 0.40 to 0.59, *p* < 0.05) with PM_2.5_ and NO_2_ concentration at ‘home indoor’ microenvironments, NO_2_ in ‘others indoor’ and ‘transit’ microenvironments, O_3_ at ‘office indoor’ microenvironments, and ambient PM_2.5_ and O_3_ concentration. There was also a weak but statistically significant correlation (i.e., 0.20 to 0.33, *p* < 0.05) of TIAR_combine_ with time spent at ‘home indoor’ microenvironments, PM_2.5_ and O_3_ at ‘others indoor’ microenvironments, PM_2.5_, and NO_2_ and O_3_ at ‘outdoor’ microenvironments. These key contributors of intra-individual variability indicate that individual has great potential to reduce their health damage related to combined exposure to mixed particle and gaseous pollutants.

## 4. Discussion

To our best knowledge, this study is new in that is quantifies the health risk for combined personal PM_2.5_, NO_2_ and O_3_ exposure concentrations across different urban microenvironments, including ‘home indoor’, ‘office indoor’, ‘others indoor’, ‘outdoor’ and ‘transit’ microenvironments. Because health risk intensities are greater than 1 in ‘others indoor’, ‘outdoor’ and ‘transit’ microenvironments, these microenvironments contribute disproportionately to the daily health risk for combined personal PM_2.5_, NO_2_ and O_3_ exposure concentrations. This indicates that there are potentials to reduce individual’s daily health damage related to air pollution by minimizing exposure time in those microenvironments. The highest health risk intensity in ‘outdoor’ microenvironments indicates that sensitive people should avoid outdoor exposure for a longer time during high air pollution episode days. This is because the outdoor air quality in Hong Kong is contributed to by both local emission sources (e.g., on-road vehicles, marine vessels, and power plants) and regional air pollution [33,34]. People can use smart technology to check their health risk associated with outdoor air pollution, such as the Hong Kong AQHI App (Environmental Protection Department, Hong Kong) [32], Personalized Real-Time Air Quality Informatics System for Exposure—Hong Kong (PRAISE-HK) App (Hong Kong University of Science and Technology, Hong Kong) [50], AirVisual App (IQAir, Goldach, Switzerland) [51], Air Quality Index, Pollen and Fires—BreezoMeter App (BreezoMeter, Haifa, Israel) [52], Air Matters App (Air Matters Network Pty Ltd, Goldach, Switzerland) [53], ZephAir App (United States Department of State, Washington DC, USA) [54] and Plume Labs Air Quality App (Plume Labs, Paris, France) [55]. For example, the PRAISE-HK app offers real-time and forecasted high-resolution (hourly) health risk information that is related to the air pollution at a street-by-street level [50]. However, our recent study demonstrated that the long-term trend in AR for a mixture of outdoor PM_10_, NO_2_, O_3,_ and SO_2_ concentrations from 2000 to 2018 has decreased over Hong Kong because of the various emission control measures implemented by the Hong Kong government [33].

The longitudinal study design of the study provided an opportunity to quantify the inter- and intra-individual variability and the contributors of variability in terms of daily health risk. The results demonstrated that inter- and intra- individual variability in TIAR_combine_ is almost equally important. Among the identified key contributors for intra-individual variability, PM_2.5_, NO_2_ and O_3_ concentration in ‘home indoor’, O_3_ in ‘office indoor’ and ambient PM_2.5_ concentrations were significantly correlated with TIAR_combine_ by a correlation coefficient, r > 0.50. Thus, by reducing indoor sources and infiltrated ambient concentrations of PM_2.5_, NO_2_ and O_3_ in the home, O_3_ concentration at the office, and exposure to ambient PM_2.5_ concentrations, people can reduce health risk in their daily life, such as hospital admissions and emergency room visits due to respiratory and cardiovascular diseases. People can reduce the infiltrated ambient concentration at home by changing ventilation operation (i.e., reducing window opening duration, air-condition on) and using an air purifier (i.e., high-efficiency particulate air filter) [56,57,58,59]. Exposure concentration at home can be reduced from indoor sources by reducing cooking duration, not smoking inside the home, using an exhaust fan during and after cooking and using an electric stove [59,60,61,62]. At the office, people can reduce the indoor emission of O_3_ by not using the photocopier or printer and using an air purifier that produces O_3_ [63,64,65,66]. Thus, this study argues that current air quality management strategies focusing on single pollution emission sources and ambient air quality are not sufficient for effectively mitigating the public health risk of air pollution. Intra-individual variability in TIAR shows how health risk changes with changes in daily exposure concentrations and an individual’s activities. People can use PRAISE-HK app, that provides personalized information on air pollution exposure and health risk, to check their daily health risk and examine the effects of different interventions by modifying their daily activities and lifestyle to reduce their daily health risk [50]. This study provides an insight into the importance of capturing the day-to-day fluctuation in health risk of a mixture of air pollutants within an individual in future epidemiological studies. This study is new in terms of assessing the inter- and intra-individual variability and the key contributors affecting variability in the daily time-integrated health risk for combined personal exposure to particle and gaseous pollutants.

With the recent technological advancement, we measured high-resolution (1 min) air quality concentrations simultaneously for both particle and gaseous pollutants using an integrated portable sensor across the various microenvironments encountered by the participants. High-resolution personal exposure concentrations and time-location patterns across the various microenvironments provided a better representation of each microenvironment for the health risk of combined personal PM_2.5_, NO_2_ and O_3_ exposure concentrations [20]. Overall, the method based on an epidemiological AR model has been proved to be effective in quantifying the health risk for combined personal PM_2.5_, NO_2_ and O_3_ exposure concentrations across various urban microenvironments. This method provides a valuable reference that can be applied in other cities of the world to estimate personal health risk for a mixture of pollutants across the microenvironments.

The study has some limitations. The study subject sample was small because of: (1) the cost of integrated portable sensors; and (2) participant’s burdens to carry the 15 kg portable sensors all the time with them during the measurement campaign, which prevented the implementation of a large number of samples. Our study subjects were only limited to those who were affiliated to the university (i.e., students, faculty members, and staff). Hence, the results may not be representative of the people who are housewives, unemployed, retired or employed in refinery, chemical plants, and restaurants.

## 5. Conclusions

The exposure error of gaseous pollutants (i.e., NO_2_ and O_3_) based on either a fixed-site monitor or residence location as a surrogate indicates the importance of real world personal exposure concentration measurements with a daily active lifestyle. This study is new in terms of quantifying the health risk for combined personal PM_2.5_, NO_2_ and O_3_ exposure concentrations based on an epidemiological model for respiratory and cardiovascular-related hospitalization across five different microenvironments. Despite a small fraction of time spent, ‘others indoor’, ‘outdoor’ and ‘transit’ microenvironments contribute disproportionately to the daily health risk. Thus, there are potential ways to reduce an individual’s daily health risk related to air pollution by minimizing their exposure time in these microenvironments. The inter- and intra-individual variability in daily time-integrated health risk for PM_2.5_ was substantially different from the gaseous pollutants. The daily time-integrated health risk for combined PM_2.5_, NO_2_, and O_3_ (TIAR_combine_) varied by a factor up to 2.5 for a given person across measured days. Several factors were identified to be significantly correlated with daily TIAR_combine_, with the top five factors including the PM_2.5_, NO_2_ and O_3_ concentrations at ‘home indoor’ microenvironments, O_3_ concentrations at ‘office indoor’ microenvironments and ambient PM_2.5_ concentrations. The results on the key contributors of variability in the daily personal health risk for combined PM_2.5_, NO_2,_ and O_3_ exposure concentrations can be used to guide developing strategies for reducing an individual’s health effects of air pollution. This study highlights the importance of individual health risk based on real-world exposure to a mixture of air pollutants that policymakers can prioritize in future policy development. This study provides a reference for the method that can be applied in other cities of the world to quantify and evaluate the health risk for combined personal exposure to a mixture of pollutants.

## Figures and Tables

**Table 1:** Daily time spent (hour) across the selected microenvironments (N = 106).

Microenvironment	Min	P_25_	Median	Mean	P_75_	Max	SD
Home indoor	9.3	12.4	14.5	15.7	18.3	24.0	4.1
Office indoor	0	1.4	6.0	5.7	9.2	13.9	4.1
Others indoor	0	0.1	0.2	1.1	1.4	7.5	1.7
Outdoor	0	0.1	0.4	0.6	0.8	4.6	0.7
Transit	0	0	0.7	0.9	1.5	5.8	1.0

Definition of abbreviations: Min, Minimum; P_25_, the 25th percentile of the distribution, P_75_, the 75th percentile of the distribution; Max, maximum; SD, standard deviation.

**Table 2:** Proportion of inter- and intra-individual variability of time-integrated short-term health risk for each individual and combined pollutants.

Pollutants ^a^	$\mathrm{Inter}-\mathrm{Individual}\;\mathrm{Variance},\;\sigma_{\mathrm{inter}}^{2}\;(\%\;\mathrm{of}\;\mathrm{Total})$	$\mathrm{Intra}-\mathrm{Individual}\;\mathrm{Variance},\;\sigma_{\mathrm{intra}}^{2}\;(\%\;\mathrm{of}\;\mathrm{Total})$
TIAR_PM2.5_	22	78
TIAR_NO2_	54	46
TIAR_O3_	40	60
TIARcombine ^b^	53	47

Notes: TIAR = time-integrated health risk; ICC = $\sigma_{\mathrm{inter}}^{2}/(\sigma_{\mathrm{inter}}^{2}+\sigma_{\mathrm{intra}}^{2})$, indicates proportion of the total variations attributed to inter-individual variance. ^a^ log-transformed to ensure the normal distribution of residuals. ^b^ AR_combine_ = AR_PM2.5_ + AR_NO2_ + AR_O3._

**Table 3:** Correlation between time-integrated health risk of combined PM_2.5_, NO_2_ and O_3_ exposure concentrations (TIAR_combine_) and selected variables.

Variables ^a^	Participant Wise Average TIAR_combine_		Daily TIAR_combine_	
	Correlation Coefficient (r)	*p*-Value	Correlation Coefficient (r)	*p*-Value
Time spent at home indoor	−0.09	0.70	0.20	0.04
Time spent at office indoor	0.14	0.54	−0.02	0.84
Time spent at others indoor	−0.36	0.12	−0.07	0.54
Time spent at outdoor	0.05	0.84	0.17	0.13
Time spent at transit	0.41	0.09	0.17	0.15
PM_2.5_ (µg/m^3^) at home indoor	0.48	0.03	0.51	<0.001
PM_2.5_ (µg/m^3^) at office indoor	0.03	0.89	0.16	0.15
PM_2.5_ (µg/m^3^) at others indoor	0.45	<0.05	0.27	0.01
PM_2.5_ (µg/m^3^) at outdoor	0.40	0.07	0.33	0.002
PM_2.5_ (µg/m^3^) at transit	0.27	0.27	0.19	0.09
NO_2_ (µg/m^3^) at home indoor	0.60	0.004	0.56	<0.001
NO_2_ (µg/m^3^) at office indoor	0.12	0.60	0.15	0.18
NO_2_ (µg/m^3^) at others indoor	0.51	0.02	0.46	<0.001
NO_2_ (µg/m^3^) at outdoor	0.24	0.30	0.33	0.002
NO_2_ (µg/m^3^) at transit	0.46	0.06	0.40	<0.001
O_3_ (µg/m^3^) at home indoor	0.74	<0.001	0.76	<0.001
O_3_ (µg/m^3^) at office indoor	0.52	0.02	0.51	<0.001
O_3_ (µg/m^3^) at others indoor	0.29	0.22	0.22	0.05
O_3_ (µg/m^3^) at outdoor	0.14	0.53	0.28	0.01
O_3_ (µg/m^3^) at transit	−0.08	0.76	−0.06	0.61
Ambient PM_2.5_ (µg/m^3^)	0.56	0.01	0.59	<0.001
Ambient NO_2_ (µg/m^3^)	0.04	0.86	0.06	0.52
Ambient O_3_ (µg/m^3^)	0.07	0.77	0.41	<0.001

^a^ Variables were averaged daily and participant wise to analyze correlation with daily TIAR_combine_ and participant-wisely average TIAR_combine_, respectively.

## Data Availability

The data presented in this study are available on request to the corresponding author.

## Supplementary Materials

The following supporting information can be downloaded at: https://www.mdpi.com/article/10.3390/ijerph19010565/s1, File S1.

## References

[1] WHO (2016). WHO Global Urban Ambient Air Pollution Database (Update 2016), World Health Organization. https://www.who.int/airpollution/data/cities-2016/en/.

[2] USEPA (2019). Integrated Science Assessment for Particulate Matter.

[3] USEPA (2016). Integrated Science Assessment for Oxides of Nitrogen-Health Criteria.

[4] USEPA (2019). Integrated Science Assessment for Ozone and Related Photochemical Oxidants (External Review Draft).

[5] Li S., Batterman S., Wasilevich E., Wahl R., Wirth J., Su F.-C., Mukherjee B. (2011). Association of daily asthma emergency department visits and hospital admissions with ambient air pollutants among the pediatric Medicaid population in Detroit: Time-series and time-stratified case-crossover analyses with threshold effects. Environ. Res..

[6] Dauchet L., Hulo S., Cherot-Kornobis N., Matran R., Amouyel P., Edmé J.-L., Giovannelli J. (2018). Short-term exposure to air pollution: Associations with lung function and inflammatory markers in non-smoking, healthy adults. Environ. Int..

[7] Myung W., Lee H., Kim H. (2019). Short-term air pollution exposure and emergency department visits for amyotrophic lateral sclerosis: A time-stratified case-crossover analysis. Environ. Int..

[8] Tam W.W.S., Wong T.W., Wong A.H. (2015). Association between air pollution and daily mortality and hospital admission due to ischaemic heart diseases in Hong Kong. Atmos. Environ..

[9] Tam W.W.S., Wong T.W., Ng L., Wong S.Y.-S., Kung K.K.L., Wong A.H.S. (2014). Association between Air Pollution and General Outpatient Clinic Consultations for Upper Respiratory Tract Infections in Hong Kong. PLoS ONE.

[10] Habre R., Coull B., Moshier E., Godbold J., Grunin A., Nath A., Castro W., Schachter N., Rohr A., Kattan M. (2014). Sources of indoor air pollution in New York City residences of asthmatic children. J. Expo. Sci. Environ. Epidemiol..

[11] Che W., Frey H.C., Li Z., Lao X., Lau A.K.H. (2018). Indoor Exposure to Ambient Particles and Its Estimation Using Fixed Site Monitors. Environ. Sci. Technol..

[12] Hoek G., Beelen R., De Hoogh K., Vienneau D., Gulliver J., Fischer P., Briggs D. (2008). A review of land-use regression models to assess spatial variation of outdoor air pollution. Atmos. Environ..

[13] Eeftens M., Beelen R., de Hoogh K., Bellander T., Cesaroni G., Cirach M., Declercq C., Dėdelė A., Dons E., de Nazelle A. (2012). Development of Land Use Regression Models for PM2.5, PM2.5 Absorbance, PM10 and PM coarse in 20 european study areas; results of the ESCAPE project. Environ. Sci. Technol..

[14] Zhang J.J., Sun L., Barrett O., Bertazzon S., Underwood F.E., Johnson M. (2015). Development of land-use regression models for metals associated with airborne particulate matter in a North American city. Atmos. Environ..

[15] Chen L., Bai Z., Kong S., Han B., You Y., Ding X., Du S., Liu A. (2010). A land use regression for predicting NO_2_ and PM10 concentrations in different seasons in Tianjin region, China. J. Environ. Sci..

[16] Mazaheri M., Clifford S., Yeganeh B., Viana M., Rizza V., Flament R., Buonanno G., Morawska L. (2018). Investigations into factors affecting personal exposure to particles in urban microenvironments using low-cost sensors. Environ. Int..

[17] Koehler K., Good N., Wilson A., Mölter A., Moore B.F., Carpenter T., Peel J.L., Volckens J. (2019). The Fort Collins commuter study: Variability in personal exposure to air pollutants by microenvironment. Indoor Air.

[18] Ma J., Tao Y., Kwan M.-P., Chai Y. (2020). Assessing Mobility-Based Real-Time Air Pollution Exposure in Space and Time Using Smart Sensors and GPS Trajectories in Beijing. Ann. Am. Assoc. Geogr..

[19] Watson A.Y., Bates R.R., Kennedy D. (1988). Assessment of Human Exposure to Air Pollution: Methods, Measurements, and Models. https://www.ncbi.nlm.nih.gov/books/NBK218147/.

[20] Van Ryswyk K., Wheeler A.J., Wallace L., Kearney J., You H., Kulka R., Xu X. (2014). Impact of microenvironments and personal activities on personal PM2.5 exposures among asthmatic children. J. Expo. Sci. Environ. Epidemiol..

[21] Kolluru S.S.R., Patra A.K., Sahu S.P. (2018). A comparison of personal exposure to air pollutants in different travel modes on national highways in India. Sci. Total Environ..

[22] Yang F., Lau C.F., Tong V.W.T., Zhang K.K., Westerdahl D., Ng S., Ning Z. (2018). Assessment of personal integrated exposure to fine particulate matter of urban residents in Hong Kong. J. Air Waste Manag. Assoc..

[23] Jahn H.J., Kraemer A., Chen X.-C., Chan C.-Y., Engling G., Ward T.J. (2013). Ambient and personal PM2.5 exposure assessment in the Chinese megacity of Guangzhou. Atmos. Environ..

[24] Tunno B.J., Dalton R., Michanowicz D.R., Shmool J.L.C., Kinnee E., Tripathy S., Cambal L., Clougherty J.E. (2016). Spatial patterning in PM_2.5_ constituents under an inversion-focused sampling design across an urban area of complex terrain. Expo. Sci. Environ. Epidemiol..

[25] Li N., Xu C., Liu Z., Li N., Chartier R., Chang J., Wang Q., Wu Y., Li Y., Xu D. (2020). Determinants of personal exposure to fine particulate matter in the retired adults—Results of a panel study in two megacities, China. Environ. Pollut..

[26] Chen X.-C., Jahn H.J., Ward T.J., Ho H.C., Luo M., Engling G., Kraemer A. (2020). Characteristics and determinants of personal exposure to PM2.5 mass and components in adult subjects in the megacity of Guangzhou, China. Atmos. Environ..

[27] Chen X.-C., Ward T.J., Cao J.-J., Lee S.-C., Chow J.C., Lau G.N., Yim S.H.L., Ho K.-F. (2018). Determinants of personal exposure to fine particulate matter (PM2.5) in adult subjects in Hong Kong. Sci. Total Environ..

[28] Sanchez M., Milà C., Sreekanth V., Balakrishnan K., Sambandam S., Nieuwenhuijsen M., Kinra S., Marshall J.D., Tonne C. (2020). Personal exposure to particulate matter in peri-urban India: Predictors and association with ambient concentration at residence. J. Expo. Sci. Environ. Epidemiol..

[29] Lee K., Bartell S.M., Paek M. (2004). Interpersonal and daily variability of personal exposures to nitrogen dioxide and sulfur dioxide. J. Expo. Sci. Environ. Epidemiol..

[30] Grivas G., Dimakopoulou K., Samoli E., Papakosta D., Karakatsani A., Katsouyanni K., Chaloulakou A. (2017). Ozone exposure assessment for children in Greece-Results from the RESPOZE study. Sci. Total Environ..

[31] Wong T.W., Tam W., Yu I.T.S., Lau A., Pang S.W., Wong A.H. (2013). Developing a risk-based air quality health index. Atmos. Environ..

[32] HKEPD Air Quality Health Index-Frequently Asked Questions. Environmental Protection Department, Hong Kong, 2019. http://www.aqhi.gov.hk/en/what-is-aqhi/faqs.html#e_05.

[33] Hossain S., Frey H.C., Louie P.K., Lau A.K. (2021). Combined effects of increased O_3_ and reduced NO_2_ concentrations on short-term air pollution health risks in Hong Kong. Environ. Pollut..

[34] Lee S.C., Cheng Y., Ho K.F., Cao J.J., Louie P.K.-K., Chow J.C., Watson J. (2006). PM1.0 and PM2.5 characteristics in the roadside environment of Hong Kong. Aerosol Sci. Technol..

[35] Che W., Tso C.Y., Sun L., Ip D.Y., Lee H., Chao Y.H.C., Lau A.K. (2019). Energy consumption, indoor thermal comfort and air quality in a commercial office with retrofitted heat, ventilation and air conditioning (HVAC) system. Energy Build..

[36] Sun L., Wong K.C., Wei P., Ye S., Huang H., Yang F., Westerdahl D., Louie P.K., Luk C.W., Ning Z. (2016). Development and application of a next generation air sensor network for the Hong Kong marathon 2015 air quality monitoring. Sensors.

[37] Sun L., Westerdahl D., Ning Z. (2017). Development and evaluation of a novel and cost-effective approach for low-cost NO_2_ sensor drift correction. Sensors.

[38] Che W., Li A.T.Y., Frey H.C., Tang K.T.J., Sun L., Wei P., Hossain S., Hohenberger T.L., Leung K.W., Lau A.K.H. (2020). Factors affecting variability in gaseous and particle microenvironmental air pollutant concentrations in Hong Kong primary and secondary schools. Indoor Air.

[39] Hossain S., Che W., Frey H.C., Lau A.K. (2021). Factors affecting variability in infiltration of ambient particle and gaseous pollutants into home at urban environment. Build. Environ..

[40] Persily A.K. (1997). Evaluating building IAQ and ventilation with indoor carbon dioxide. ASHRAE Trans..

[41] Satish U., Mendell M.J., Shekhar K., Hotchi T., Sullivan D., Streufert S., Fisk W.J. (2012). Is CO_2_ an Indoor Pollutant? Direct Effects of Low-to-Moderate CO_2_ Concentrations on Human Decision-Making Performance. Environ. Health Perspect..

[42] Klepeis N.E. (1999). An introduction to the indirect exposure assessment approach: Modeling human exposure using microenvironmental measurements and the recent National Human Activity Pattern Survey. Environ. Health Perspect..

[43] Mazaheri M., Clifford S., Jayaratne R., Mokhtar M.A.M., Fuoco F., Buonanno G., Morawska L. (2013). School children’s personal exposure to ultrafine particles in the urban environment. Environ. Sci. Technol..

[44] Xu R. (2003). Measuring explained variation in linear mixed effects models. Stat. Med..

[45] Sahai H., Ageel M.I. (2000). The Analysis of Variance: Fixed, Random, and Mixed Models.

[46] Xu M., Sbihi H., Pan X., Brauer M. (2019). Local variation of PM2.5 and NO_2_ concentrations within metropolitan Beijing. Atmos. Environ..

[47] Squizzato S., Masiol M., Rich D.Q., Hopke P.K. (2018). PM2.5 and gaseous pollutants in New York State during 2005–2016: Spatial variability, temporal trends, and economic influences. Atmos. Environ..

[48] Baxter L.K., Clougherty J.E., Laden F., Levy J.I. (2007). Predictors of concentrations of nitrogen dioxide, fine particulate matter, and particle constituents inside of lower socioeconomic status urban homes. J. Expo. Sci. Environ. Epidemiol..

[49] Schober P., Boer C., Schwarte L.A. (2018). Correlation coefficients: Appropriate use and interpretation. Anesth. Analg..

[50] Che W., Frey H.C., Fung J.C., Ning Z., Qu H., Lo H.K., Chen L., Wong T.-W., Wong M.K., Lee O.C. (2020). PRAISE-HK: A personalized real-time air quality informatics system for citizen participation in exposure and health risk management. Sustain. Cities Soc..

[51] IQAir (2021). IQAir | The World’s Leading Air Quality App. https://www.iqair.com/air-quality-app.

[52] BreezoMeter (2021). Accurate Air Quality, Pollen & Active Fires Information|BreezoMeter. https://www.breezometer.com/.

[53] AirMatters (2021). Air Matters—A Global Air Quality Service Provider. https://air-matters.com/index.html.

[54] USDOS (2021). ZephAir App Available Now—United States Department of State. https://www.state.gov/zephair-app-available-now/.

[55] PlumeLabs (2021). Plume Labs App: Live and Forecast air Quality Data. https://plumelabs.com/en/air/.

[56] Allen R.W., Adar S.D., Avol E., Cohen M., Curl C.L., Larson T., Liu L.-J.S., Sheppard L., Kaufman J. (2012). Modeling the Residential Infiltration of Outdoor PM 2.5 in the Multi-Ethnic Study of Atherosclerosis and Air Pollution (MESA Air). Environ. Health Perspect..

[57] Zhou X., Cai J., Zhao Y., Chen R., Wang C., Zhao A., Yang C., Li H., Liu S., Cao J. (2016). Estimation of residential fine particulate matter infiltration in Shanghai, China. Environ. Pollut..

[58] Xu C., Li N., Yang Y., Li Y., Liu Z., Wang Q., Zheng T., Civitarese A., Xu D. (2017). Investigation and modeling of the residential infiltration of fine particulate matter in Beijing, China. J. Air Waste Manag. Assoc..

[59] MacNeill M., Wallace L., Kearney J., Allen R., Van Ryswyk K., Judek S., Xu X., Wheeler A. (2012). Factors influencing variability in the infiltration of PM 2.5 mass and its components. Atmos. Environ..

[60] Dėdelė A., Miškinytė A. (2016). Seasonal variation of indoor and outdoor air quality of nitrogen dioxide in homes with gas and electric stoves. Environ. Sci. Pollut. Res..

[61] Dobbin N.A., Sun L., Wallace L., Kulka R., You H., Shin T., Aubin D., St-Jean M., Singer B.C. (2018). The benefit of kitchen exhaust fan use after cooking—An experimental assessment. Build. Environ..

[62] Hu Y., Zhao B. (2020). Relationship between indoor and outdoor NO_2_: A review. Build. Environ..

[63] Huang Y., Yang Z., Gao Z. (2019). Contributions of indoor and outdoor sources to ozone in residential buildings in Nanjing. Int. J. Environ. Res. Public Health.

[64] Lee S.-C., Lam S., Fai H.K. (2001). Characterization of VOCs, ozone, and PM10 emissions from office equipment in an environmental chamber. Build. Environ..

[65] Britigan N., Alshawa A., Nizkorodov S.A. (2006). Quantification of ozone levels in indoor environments generated by ionization and ozonolysis air purifiers. J. Air Waste Manag. Assoc..

[66] Guo C., Gao Z., Shen J. (2019). Emission rates of indoor ozone emission devices: A literature review. Build. Environ..

