# Supplementary material for: Inter- and Intra-Individual Variability of Personal Health Risk of Combined Particle and Gaseous Pollutants across Selected Urban Microenvironments
Source: Int J Environ Res Public Health. 2022 Jan 5;19(1):565. doi: 10.3390/ijerph19010565 (PMC8744794; doi:10.3390/ijerph19010565)
Supplement: Supplementary file 1 [file ijerph-19-00565-s001.zip › ijerph-1488061-supplementary.pdf]

Supplementary file S1

1. Sensors for measuring personal exposure concentrations

Twenty-four hours personal exposure concentrations of $PM_{2.5}$, NO_2, O_3 and CO_2 were across the various urban microenvironment were measured using an integrated portable sensor system. Table S1 shows the specifications of the sensors using in the integrated portable system to measure personal exposure concentrations.

Table S1. Specifications of sensors used in integrated portable sensor systems to measure personal exposure concentrations.

Sensor specifications	Pollutants			
	$PM_{2.5}$	NO_2	O_3	CO_2
Concentration range	0-1000 μg/m^3	0~20 ppm	0~20 ppm	0~5000 ppm
Detection limit	1 μg/m^3	5 ppb	0.01 ppm	300 ppm
Accuracy	± 10% to calibration aerosol	±10 ppb (under normal maintenance)	±10 ppb or 15% of measured concentration	±10% of reading
Sensor technology	Optical	Electrochemical	Electrochemical	Nondispersive infrared
Manufacturer	Metone, Grants Pass, OR	Alphasense, Braintree, UK	Alphasense, Braintree, UK	Dynament, Mansfield, UK

2. CO_2 concentrations as an indicator of microenvironments

Carbon dioxide (CO_2) is produced by human exhalation as a metabolic process function (Persily, 1997). Thus, the outdoor CO_2 concentrations level is lower than the CO_2 concentrations in indoor environments because of human occupancy (Satish et al., 2012). A number of studies identified the presence or absence of any occupant in indoor areas based on CO_2 concentration (Calì et al., 2015; Jiang et al., 2016; Pedersen et al., 2017; Sun et al., 2011; Szczurek et al., 2017). Fig. S1 shows an example of temporal CO_2 concentration variation across the microenvironments visited by a participant in a day from morning to evening. Because CO_2 concentration substantially varies between indoor and outdoor

microenvironments and among different indoor microenvironments depending on the occupancy number and ventilation condition, real-time CO_2 concentration time-series were used in this study to check consistency of the recorded time-location patterns of the individual.

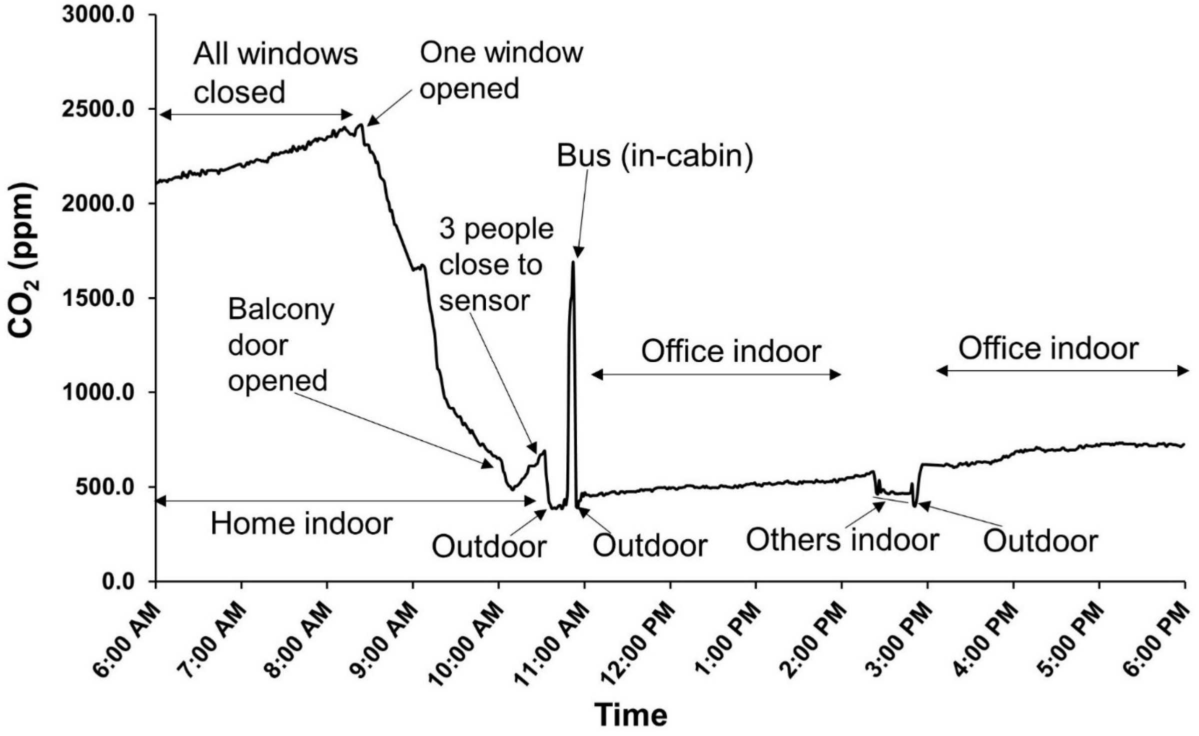

Figure S1. Example of temporal CO_2 concentration variation across the microenvironments visited by a participant in a day from morning to evening.

3. Comparison of health risk estimation between 1-minute personal exposure concentrations and 3-h moving average concentrations

Health risk (added health risk, AR) for combined personal $PM_{2.5}$, NO_2 and O_3 exposure concentrations were quantified following the study by Wong et al. (2013). In our study, AR was estimated based on continuous 1-minute personal exposure concentrations, although Wong et al. (2013) quantified AR hourly based on a 3-h moving average of ambient concentrations. This is because we quantified AR in each microenvironment across the person-days, and participants spent less than 3 hours in some of the microenvironments. Thus, 3-h moving average concentrations may cause misrepresentation of those microenvironments by averaging before

and after microenvironmental concentrations.

Percentage difference in AR estimation between 1-minute average data and 3-hour moving average concentration for each pollutant in each person-day, regardless of the microenvironments, was estimated (Cole and Altman, 2017):

$$E_p = \left| \frac{AR_{p,1min} - AR_{p,3hour}}{\frac{AR_{p,1min} + AR_{p,3hour}}{2}} \right| \times 100 \quad (1)$$

Where E_p is the percentage difference in daily average AR estimation between 1-minute average data and 3-hour moving average concentration of pollutant p. $AR_{p,1min}$ is the daily average AR estimation based on 1-minute average data of pollutant p. $AR_{p,3hour}$ is the daily average AR estimation based on 3-hour moving average concentration of pollutant p.

Fig. S2 shows the percentage difference in daily average AR estimation between 1-minute average data and 3-hour moving average concentration for each pollutant in each person-day across the participants. The results showed that the median differences of AR estimation between 1-minute exposure concentrations and 3-h moving average concentrations were less than 2% for each pollutant, regardless of the microenvironments.

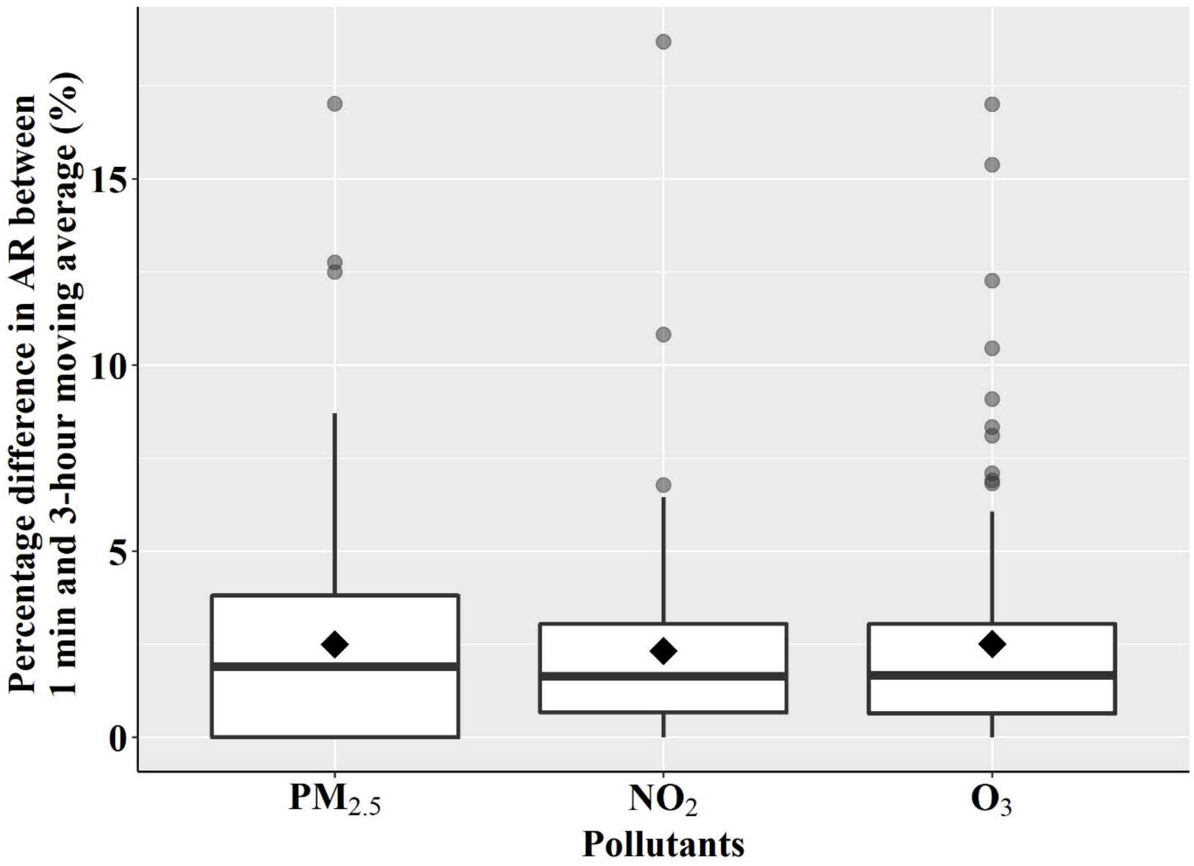

Figure S2. Percentage difference in daily average AR estimation between 1-minute average data and 3-hour moving average concentration for each pollutant in each person-day (N = 106).

4. General characteristics of the study participants

General characteristics of the study participants are provided in Table S2.

Table S2. General characteristics of the study participants (N=21).

**Characteristics**		**No. (%)**
Study subjects (N)		21
Average person-days per subject		5 days
Total person-days of measurement		106 days
Age (years)		21-60 years
Occupation		
	Postgraduate student	7 (33.3%)
	Faculty	7 (33.3%)
	Office staff	7 (33.3%)
Type of ventilation status at home		
	Open window	10 (47.6%)
	Turn on air-condition (AC)	1 (4.8%)
	Open window and turn on AC	10 (47.6%)
AC type in home		
	Window type AC	16 (76.2%)
	Split type AC	2 (9.5%)

	Both window and split AC	3 (14.3%)
Cooking stoves		
	Town gas	13 (61.9%)
	Electricity	8 (38.1%)
Cooking duration per day		
	<80 minutes	6 (28.6%)
	≥80 minutes	15 (71.4%)
Frequency of floor cleaning		
	Everyday	14 (66.7%)
	Sometimes in a week	7 (33.3%)
Floor		
	≤8	14 (66.7%)
	>8	7 (33.3%)
Occupants (person)		
	<4	14 (66.7%)
	≥4	7 (33.3%)
Smoking (by participants or family members)		No

5. Inter-and intra-individual variability in daily time spent across the selected microenvironments

To quantify variance component in daily time spent across the selected microenvironmnets, a linear mixed-effects model with only a random intercept was developed (Koehler et al., 2019; Li et al., 2020). Variance component analysis in daily time spent was performed separately for each microenvironment.

$$Y_{ijk} = \mu + b_i + \varepsilon_{ijk} \qquad (1)$$

Where Y_{ijk} is the log-transformed daily time spent in a microenvironment k at person-day j for participant i. For Office indoor microenvironment, daily time spent is not log-transformed because residuals are normally distributed without log-transformation. μ is the fixed mean (logged) daily time spent for all subjects. ε_{ijk} is the error. In linear mixed-effects models, person-specific random effect b_i assumed to be normally distributed with zero mean and variance $\sigma^2_{\mathrm{inter}}$ $\sigma^2_{\mathrm{inter}}$ (the inter-individual variability). Error ε_{ijp} is assumed to be normal distribution with zero mean and variance $\sigma^2_{\mathrm{intra}}$ (the intra- variability). The variance components for inter-and intra-individual were estimated using the method of restricted maximum likelihood (REML) (Xu,

2003).

The proportion of inter-and intra-individual variability of daily time spent in each microenvironment is given in Table S3. Intra-individual variability in daily time spent in all microenvironments ranging from 59% - 91% was higher than the inter-individual variability.

Table S3. Proportion of inter-and intra-individual variability of daily time spent in each microenvironment.

Microenvironments	Inter-individual variance, σ^2_{inter} (% of total)	Intra-individual variance, σ^2_{intra} (% of total)
Home indoor	15	85
Office indoor[a]	9	91
Others indoor	26	74
Outdoor	39	61
Transit	41	59

Notes: ICC = $\sigma^2_{inter} / (\sigma^2_{inter} + \sigma^2_{intra})$, indicates the proportion of the total variations attributed to inter-individual variance
[a]Daily time spent is not log-transformed

6. Personal exposure concentrations of $PM_{2.5}$, NO_2 and O_3 across the selected microenvironments

Table S4 presents summary statistics of personal exposure concentrations of $PM_{2.5}$, NO_2 and O_3 in each microenvironment across the participants.

Table S4. Summary statistics of personal exposure concentrations of $PM_{2.5}$, NO_2 and O_3 in each microenvironment across the participants.

Microenvironments	N (1-min)	$PM_{2.5}$		NO_2		O_3	
		Mean (μgm^{-3})	SD (μgm^{-3})	Mean (μgm^{-3})	SD (μgm^{-3})	Mean (μgm^{-3})	SD (μgm^{-3})
Home indoor	96259	20	12	33	19	31	22
Office indoor	34973	4	3	26	14	35	17
Others indoor	6661	19	32	42	33	24	20
Outdoor	3343	21	12	66	45	42	31
Transit	5355	17	16	89	94	15	15

N: 1-minute observations; SD: Standard deviation

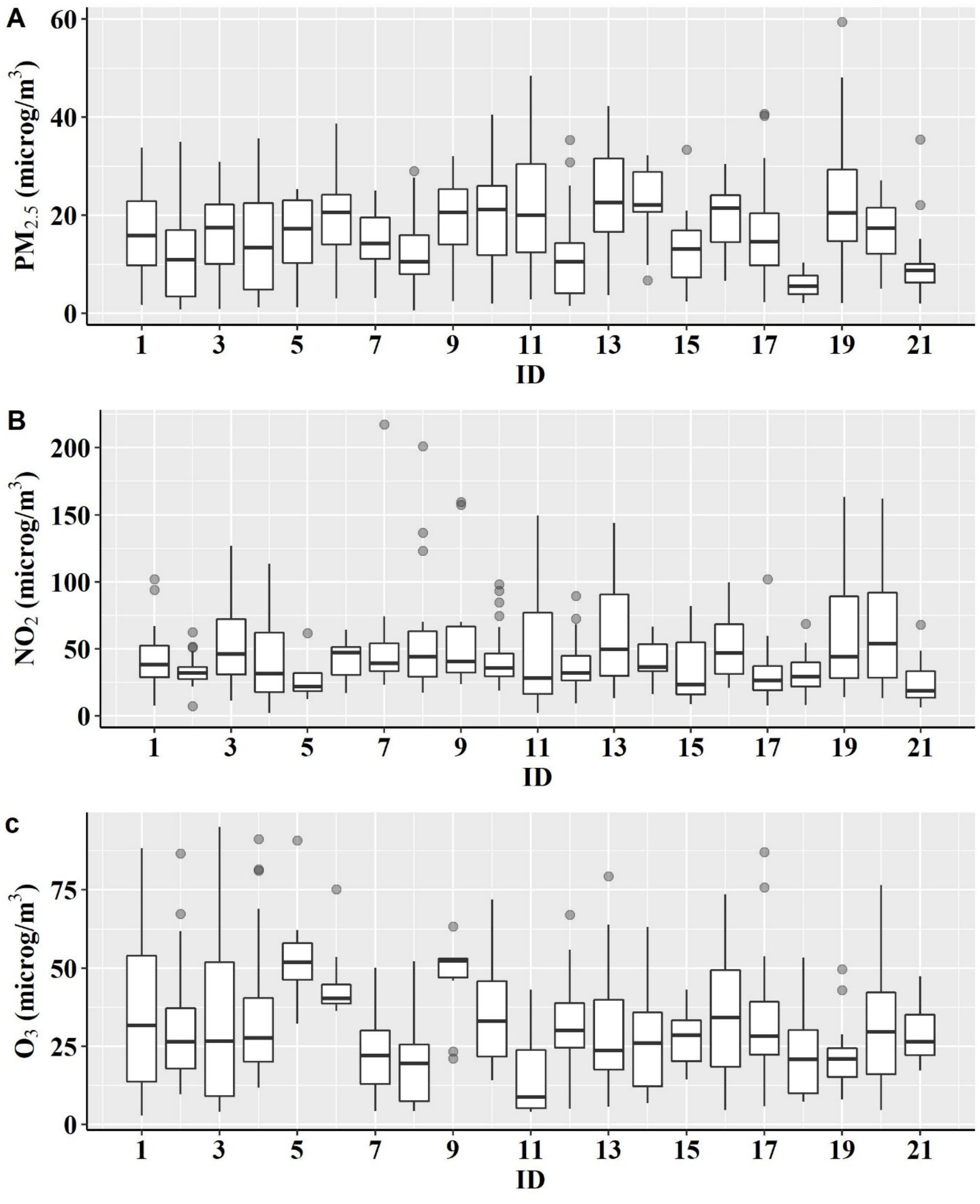

Figure S3. Distribution of personal exposure concentrations of $PM_{2.5}$, NO_2 and O_3 for each participant.

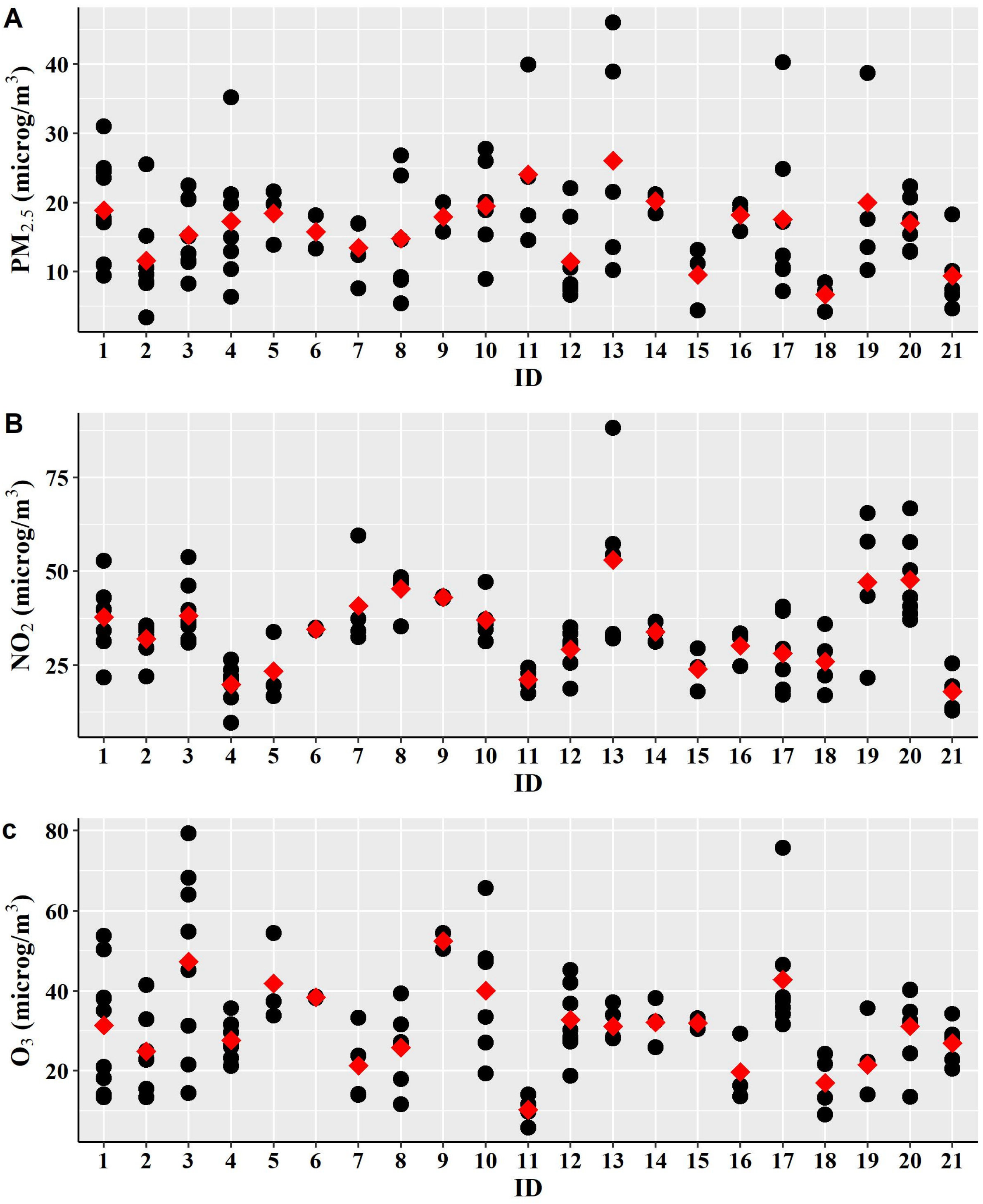

Figure S4. Temporal variation of $PM_{2.5}$, NO_2 and O_3 exposure concentrations across different person-days of the participants. Black circle represents the average concentrations in each person-day. Red square indicates mean concentration of different person-days for a participant.

7. Relationship of ambient $PM_{2.5}$, NO_2 and O_3 concentrations between Fixed-site monitor and residence-location

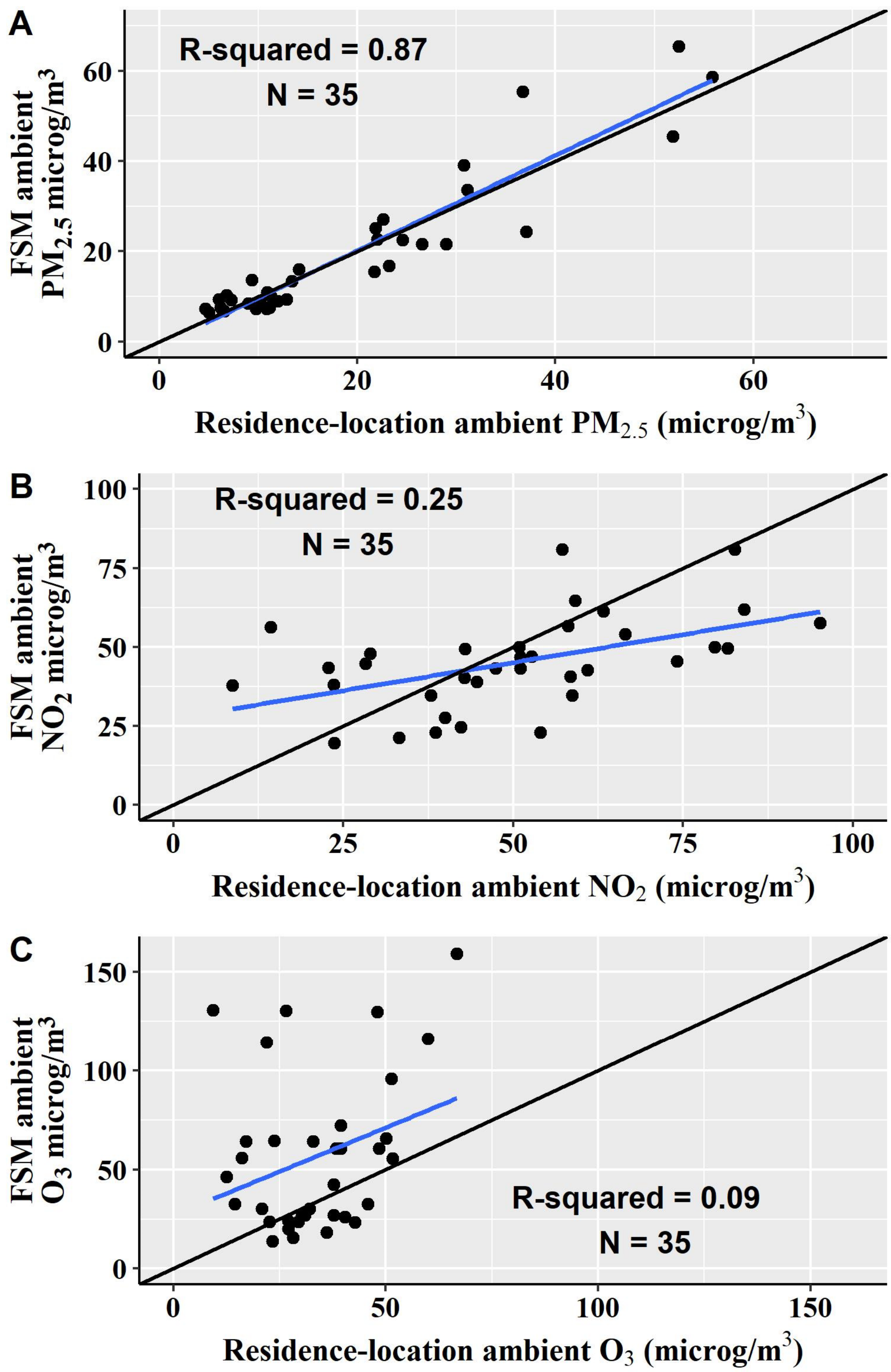

Figure S5. Relationship of daily average ambient $PM_{2.5}$, NO_2 and O_3 concentrations between Fixed-site monitor and residence-location: (A) $PM_{2.5}$, (B) NO_2 and (C) O_3. FSM denotes fixed-site monitor.

8. Time-integrated health risk for combined $PM_{2.5}$, NO_2 and O_3 exposure concentrations

Health risk and time-integrated health risk for combined $PM_{2.5}$, NO_2 and O_3 exposure

concentrations across the selected microenvironments are given in Table S5.

Table S5. Time-integrated health risk for combined $PM_{2.5}$, NO_2 and O_3 exposure concentrations across the selected microenvironments.

Microenvironment	N	Health Risk, $AR_{combine}$ (%)	Mean % time-integrated $AR_{combine}$ (SD)
		Mean (SD)	
Home indoor	106	3.45 (1.19)	64 (19)
Office indoor	86	3.02 (0.84)	27 (15)
Others indoor	82	3.82 (1.40)	6 (8)
Outdoor	85	5.35 (1.61)	5 (5)
Transit	78	5.42 (3.27)	7 (7)

N, person-days; SD, Standard deviation; $AR_{combine} = AR_{PM2.5} + AR_{NO2} + AR_{O3}$

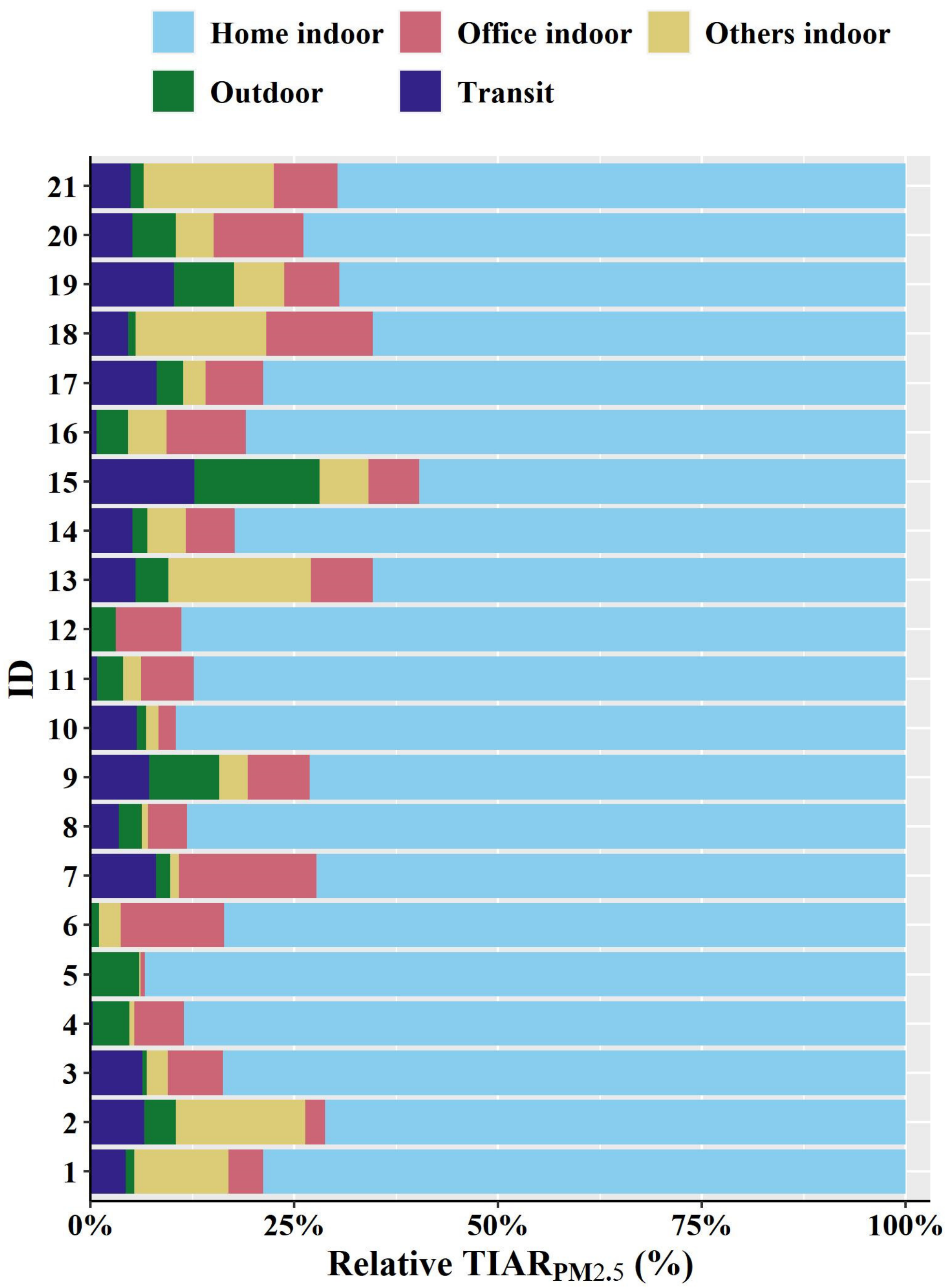

Figure S6. Variability of of the daily time-integrated health risk (TIAR) for $PM_{2.5}$ across the selected microenvironments for each participant.

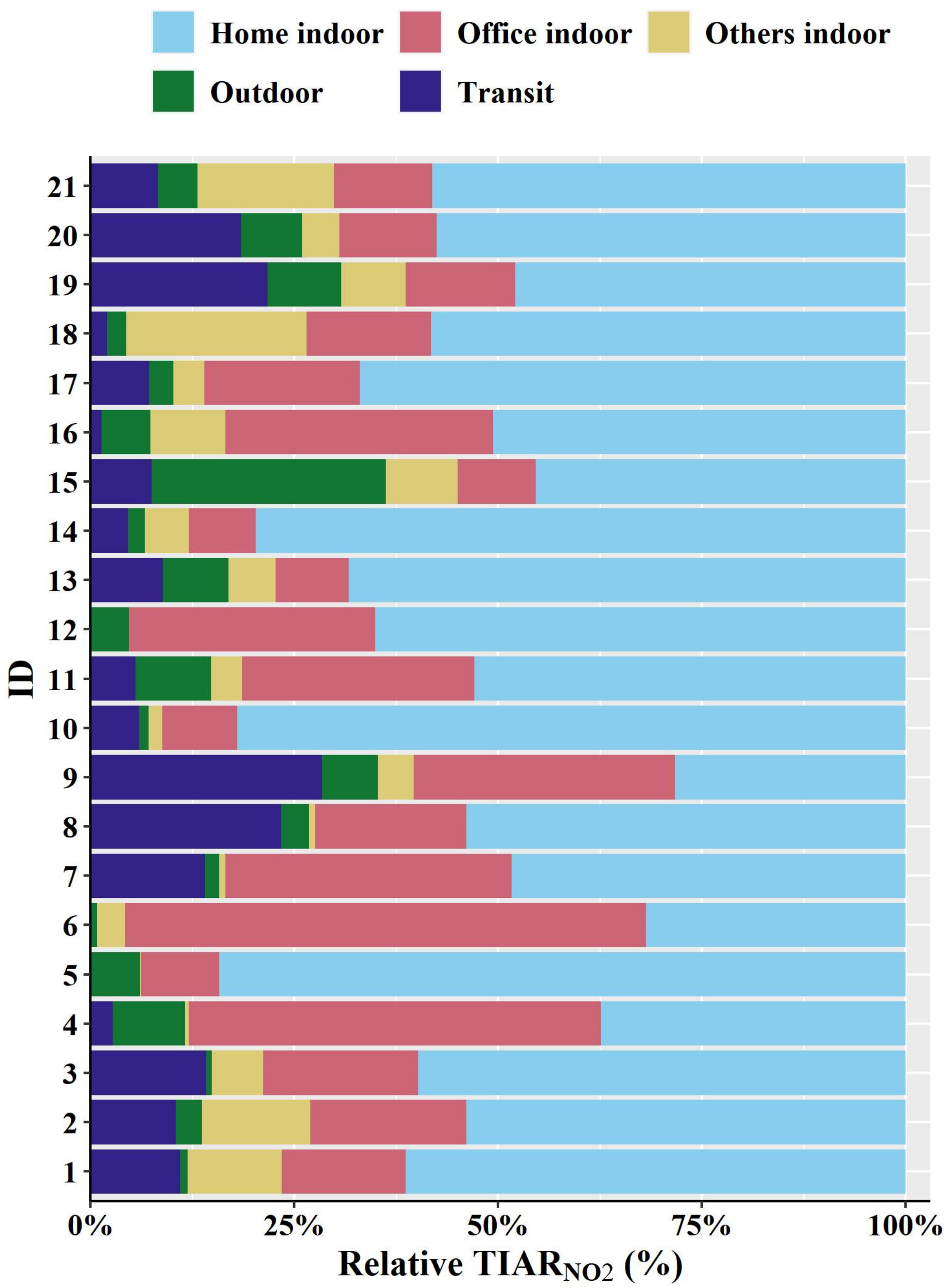

Figure S7. Variability of of the daily time-integrated health risk (TIAR) for NO_2 across the selected microenvironments for each participant.

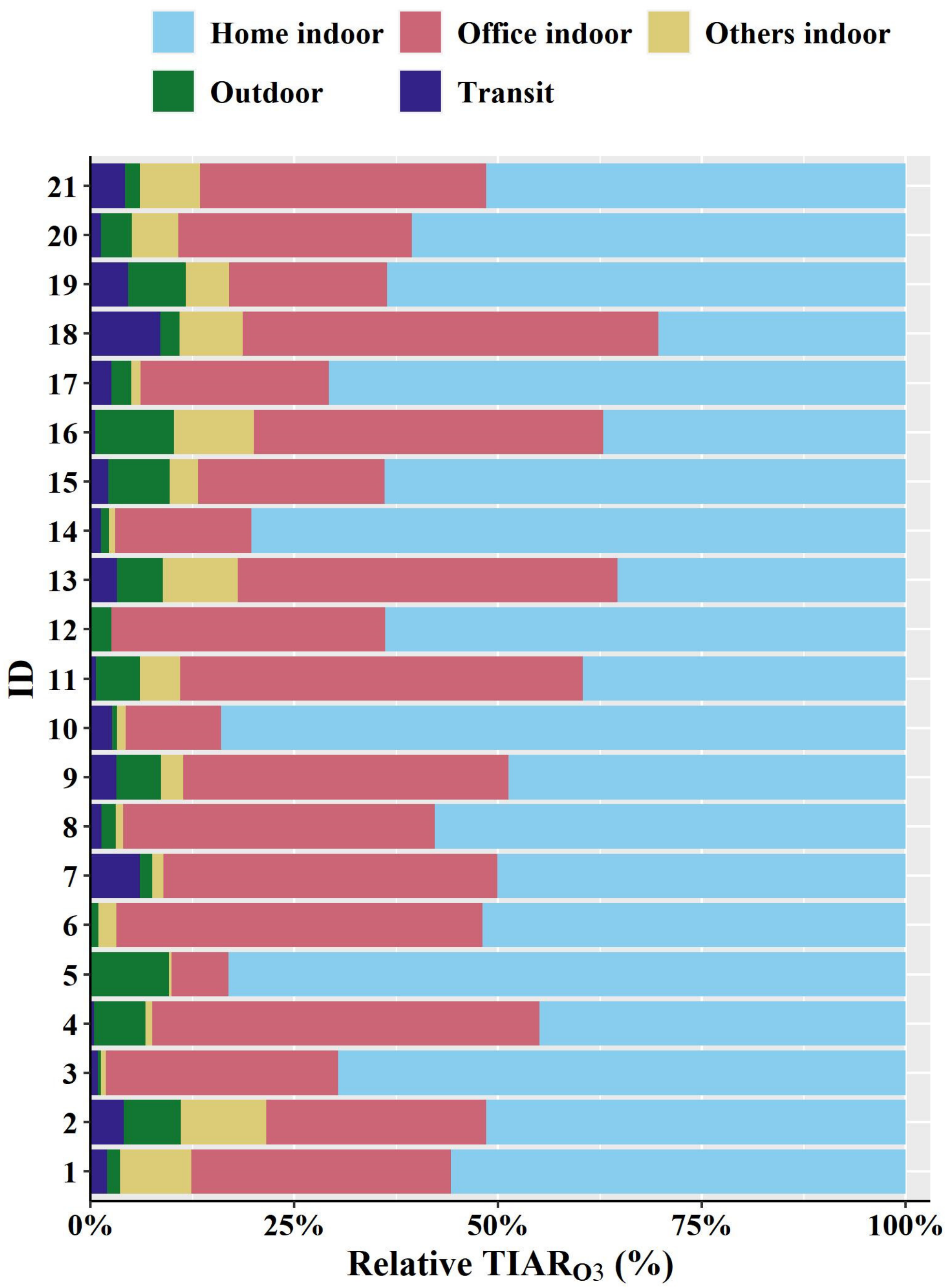

Figure S8. Variability of of the daily time-integrated health risk (TIAR) for O_3 across the selected microenvironments for each participant.

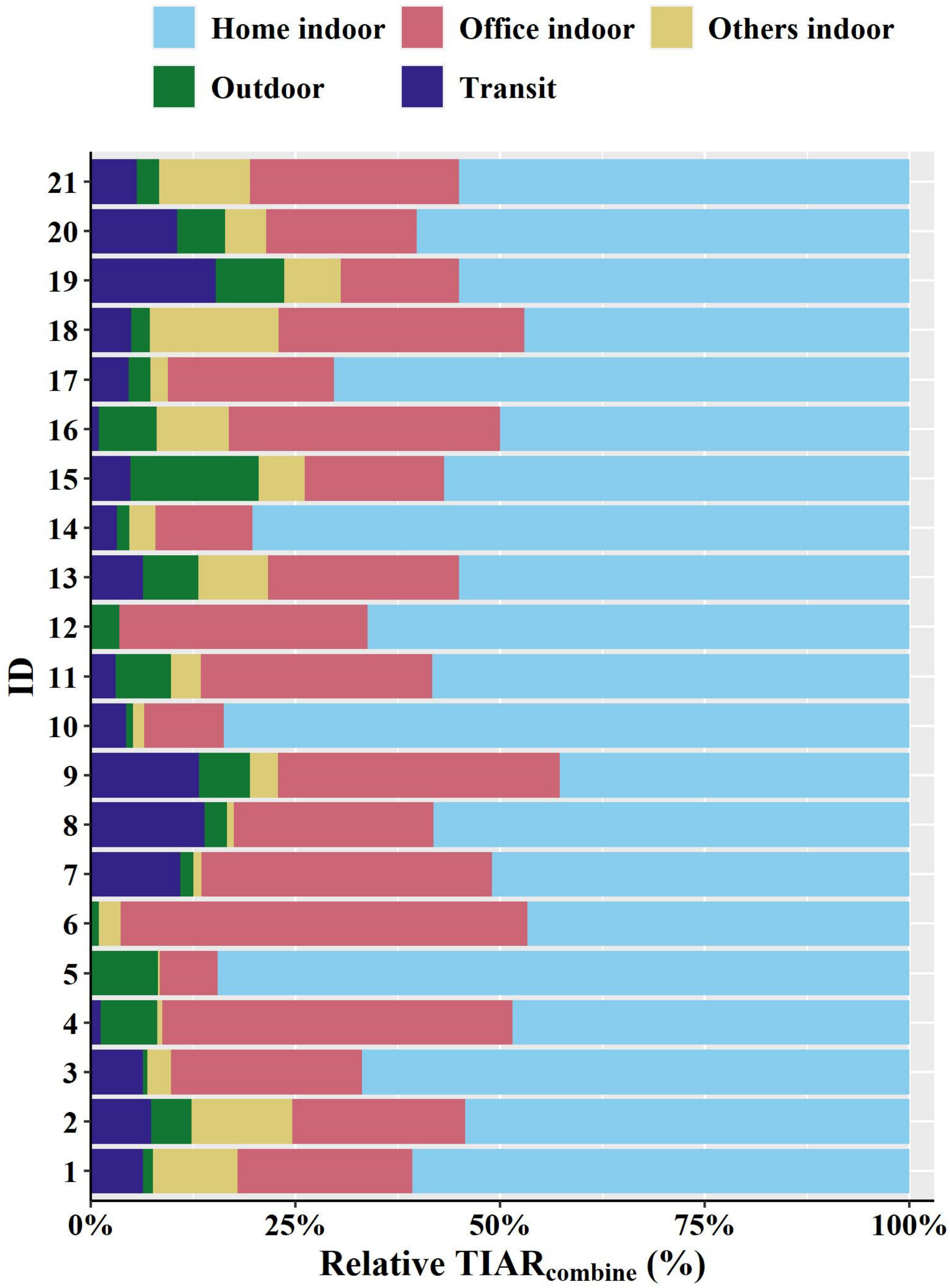

Figure S9. Variability of of the daily time-integrated health risk (TIAR) for combined $PM_{2.5}$, NO_2 and O_3 exposure concentrations across the selected microenvironments for each participant.

References

Calì, D., Matthes, P., Huchtemann, K., Streblow, R., Müller, D., 2015. CO 2 based occupancy detection algorithm: Experimental analysis and validation for office and residential buildings. Build. Environ. 86, 39–49. https://doi.org/10.1016/j.buildenv.2014.12.011

Cole, T.J., Altman, D.G., 2017. Statistics Notes: What is a percentage difference? BMJ 358, 3663. https://doi.org/10.1136/bmj.j3663

Jiang, C., Masood, M.K., Soh, Y.C., Li, H., 2016. Indoor occupancy estimation from carbon dioxide concentration. Energy Build. 131, 132–141. https://doi.org/10.1016/j.enbuild.2016.09.002

Pedersen, T.H., Nielsen, K.U., Petersen, S., 2017. Method for room occupancy detection based on trajectory of indoor climate sensor data. Build. Environ. 115, 147–156. https://doi.org/10.1016/j.buildenv.2017.01.023

Persily, A.K., 1997. Evaluating building IAQ and ventilation with indoor carbon dioxide. ASHRAE Trans. 103, 193–204.

Satish, U., Mendell, M.J., Shekhar, K., Hotchi, T., Sullivan, D., 2012. Concentrations on Human Decision-Making Performance. Environ. Health Perspect. 120, 1671–1678.

Sun, Z., Wang, S., Ma, Z., 2011. In-situ implementation and validation of a CO2-based adaptive demand-controlled ventilation strategy in a multi-zone office building. Build. Environ. 46, 124–133. https://doi.org/10.1016/j.buildenv.2010.07.008

Szczurek, A., Maciejewska, M., Pietrucha, T., 2017. Occupancy determination based on time series of CO 2 concentration, temperature and relative humidity. Energy Build. 147, 142–154. https://doi.org/10.1016/j.enbuild.2017.04.080

Wong, T.W., Tam, W.W.S., Yu, I.T.S., Lau, A.K.H., Pang, S.W., Wong, A.H.S., 2013. Developing a risk-based air quality health index. Atmos. Environ. 76, 52–58. https://doi.org/10.1016/J.ATMOSENV.2012.06.071
